# Surface‐assisted laser desorption/ionization mass spectrometry imaging: A review

**DOI:** 10.1002/mas.21670

**Published:** 2020-11-10

**Authors:** Wendy H. Müller, Alexandre Verdin, Edwin De Pauw, Cedric Malherbe, Gauthier Eppe

**Affiliations:** ^1^ Mass Spectrometry Laboratory, MolSys Research Unit, Chemistry Department University of Liège Liège Belgium

**Keywords:** desorption/ionization, imaging, mass spectrometry, nanomaterials, SALDI, small molecules

## Abstract

In the last decades, surface‐assisted laser desorption/ionization mass spectrometry (SALDI‐MS) has attracted increasing interest due to its unique capabilities, achievable through the nanostructured substrates used to promote the analyte desorption/ionization. While the most widely recognized asset of SALDI‐MS is the untargeted analysis of small molecules, this technique also offers the possibility of targeted approaches. In particular, the implementation of SALDI‐MS imaging (SALDI‐MSI), which is the focus of this review, opens up new opportunities. After a brief discussion of the nomenclature and the fundamental mechanisms associated with this technique, which are still highly controversial, the analytical strategies to perform SALDI‐MSI are extensively discussed. Emphasis is placed on the sample preparation but also on the selection of the nanosubstrate (in terms of chemical composition and morphology) as well as its functionalization possibilities for the selective analysis of specific compounds in targeted approaches. Subsequently, some selected applications of SALDI‐MSI in various fields (i.e., biomedical, biological, environmental, and forensic) are presented. The strengths and the remaining limitations of SALDI‐MSI are finally summarized in the conclusion and some perspectives of this technique, which has a bright future, are proposed in this section.

**Acronyms**
9‐AA: 9‐aminoacridine
AgLDI: silver‐assisted laser desorption/ionization
AgNPET: silver nanoparticle‐enhanced target
AMP: adenosine monophosphate
ATP: adenosine triphosphate
AuNPET: gold nanoparticle‐enhanced target
BP: benzylpyridinium
CALDI: cation‐assisted laser desorption/ionization
Cer: ceramides
CHCA: apha‐cyano‐4‐hydroxycinnamic acid
CPA: cyclic phosphatidic acid
DAN: 1,5‐diaminonaphthalene
DESI: desorption electrospray ionization
DG: diglycerides
DHB: 2,5‐dihydroxybenzoic acid
DIOM: desorption ionization on mesoporous silicate
DIOS: desorption/ionization on silicon
DIUTHAME: desorption ionization using through‐hole alumina membrane
DNA: deoxyribonucleic acid
ESI: electrospray ionization
FA: fatty acid
FFPE: formalin fixed paraffin embedded
FLT: 3’‐deoxy‐3’‐fluorothymidine
FLT‐MP: FLT monophosphate
FT‐ICR: Fourier‐transform ion cyclotron resonance
GALDI: geomatrix‐assisted laser desorption/ionization
GALDI: gold‐assisted laser desorption/ionization
GALDI: graphene‐assisted laser desorption/ionization
GALDI: graphene oxide assisted laser desorption/ionization
GALDI: graphite‐assisted laser desorption/ionization
GO: graphene oxide
GPCp: glycerophosphorylcholine phosphodiesterase
H&E: haematoxylin and eosin
IMP: inosine monophosphate
ITO: indium tin oxide
IUPAC: International Union of Pure and Applied Chemistry
LDI: laser desorption/ionization
LPE: lysophosphatidyl ethanolamine
MALDI: matrix‐assisted laser desorption/ionization
MCPA: 2‐methyl‐4‐chlorophenoxyacetic acid
MELDI: material‐enhanced laser desorption/ionization
MELDI: matrix‐enhanced laser desorption/ionization
ME‐NIMS: matrix‐enhanced nanostructure initiator mass spectrometry
ME‐SALDI: matrix‐enhanced surface‐assisted laser desorption/ionization
Mf‐MELDI: matrix‐free material‐enhanced laser desorption/ionization
MILDI: matrix‐implanted laser desorption/ionization
MILDI: matrix implantation laser desorption/ionization
MS: mass spectrometry
MSI: mass spectrometry imaging
MUC1: Mucin1
NALDI: nano‐assisted laser desorption/ionization
NALDI: nanomaterial‐assisted laser desorption/ionization
NALDI: nanoparticle‐assisted laser desorption/ionization
NALDI: nanostructure‐assisted laser desorption/ionization
NALDI: nanowire‐assisted laser desorption/ionization
Nano‐PALDI: nanoparticle‐assisted laser desorption/ionization
NAPA‐LDI: nanopost array laser desorption/ionization
NIMS: nanostructure‐initiator mass spectrometry
NIMS: nanostructure imaging mass spectrometry
nPALDI: nanoparticle‐assisted laser desorption/ionization
NPs: nanoparticles
NPs‐ALDI: nanoparticle‐assisted laser desorption/ionization
OCT: optimal cutting temperature
PA: phosphatidic acid
PC: phosphatidylcholine
PE: phosphatidylethanolamine
PHO‐S: phosphoethanolamine
PI: phosphatidyl inositol
PS: phosphatidylserine
RNA: ribonucleic acid
SA: sinapinic acid
SALDI: surface‐assisted laser desorption/ionization
SELDI: surface‐enhanced laser desorption/ionization
SERS: surface‐enhanced Raman spectroscopy
SIMS: secondary ion mass spectrometry
SPALDI: silicon nanoparticle assisted laser desorption/ionization
SPALDI: silicon nanopowder assisted laser desorption/ionization
SPILDI: silica plate imprinting laser desorption/ionization
SP‐LDI: silica plate laser desorption/ionization
ST: sulfatides
SY: survival yield
TK1: thymidine kinase
TLC: thin layer chromatography
TOF: time of flight
UV: ultraviolet
UV‐Vis: ultraviolet‐visible

## 1 INTRODUCTION: A BRIEF HISTORY OF MASS SPECTROMETRY IMAGING

Mass spectrometry imaging (MSI) has brought a new valuable dimension in mass spectrometry (MS) data collection as, besides the detection and identification of various compounds provided by conventional MS, MSI additionally allows the visualization of the spatial localization of the analytes in complex solid samples (Chughtai & Heeren, 2010; McDonnell & Heeren, 2007).

To perform MSI analyses, the analytes have to keep a precise position in the solid sample. Several strategies can be followed to generate intact gas‐phase ions from molecules in the condensed phase (Amstalden van Hove et al., 2010; Bodzon‐Kulakowska & Suder, 2016; Chughtai & Heeren, 2010; Tsai et al., 2015). Three main ionization sources are currently used in MSI. First, the sample can be bombarded by an incident beam of charged particles. This is the case in secondary ion mass spectrometry (SIMS), which involves the bombardment of the sample surface by an energetic “primary” ion or cluster beam, leading to the sputtering of secondary species from the surface (Benninghoven et al., 1987; Fearn, 2015; Schaepe et al., 2020). Second, ionization can occur under ambient conditions (Chernetsova & Morlock, 2011; Perez et al., 2019; Wu et al., 2013; Xiao et al., 2020) through the interaction of charged microdroplets of a solvent with the sample surface, in a technique called desorption electrospray ionization (DESI) (Takats et al., 2004) or, more recently, nano‐DESI (Yin et al., 2019). A third technique consists in the irradiation of the sample by a laser in a technique called laser desorption, developed about 50 years ago (Kupka et al., 1980; Posthumus et al., 1978; Vastola & Pirone, 1968). However, the high laser power required for the laser desorption of large molecules induced their fragmentation due to an increase of their internal energy. It was not until the development of the matrix‐assisted laser desorption/ionization (MALDI) technique that intact biomolecules could be analyzed by laser desorption MS. MALDI involves a laser striking light‐absorbing molecules, called “matrices,” that (i) protect the analytes from direct laser irradiation, (ii) assist the desorption and ionization of the co‐crystallized analytes. In particular, MALDI‐MS Imaging (MALDI‐MSI), promoted by the pioneering works of Spengler (Spengler et al., 1994) and Caprioli (Caprioli et al., 1997), has become the MSI reference technique for the analysis of various high molecular weight biomolecules, opening up new opportunities in the area of molecular biology (Gessel et al., 2014) but also in plant biology (Kaspar et al., 2011) and biomedicine (Schwamborn & Caprioli, 2010). However, MALDI‐MSI also suffers from limitations. First, the quality of the matrix deposit on the sample has a significant impact on the analytical performance of the MALDI‐MSI experiment. Indeed, the heterogeneity in the analyte‐matrix co‐crystallization is responsible for the formation of hot spots leading to a lack of reproducibility (both shot‐to‐shot and sample‐to‐sample reproducibility) (Goodwin, 2012; Kaletas et al., 2009). Also, the integrity of the molecular spatial distributions may be affected by an inappropriate matrix application, which may in turn cause significant migration or delocalization of the molecules of interest (Chaurand, 2012; Fournelle et al., 2020; Römpp & Spengler, 2013), affecting the spatial resolution and/or leading to misinterpretation of the MSI results. Furthermore, the formation of matrix crystals larger than the laser spot size may also affect the spatial resolution (Kaletas et al., 2009; Phan et al., 2016). For example, DHB and CHCA matrix crystals sizes are usually comprised between 5 and 20 µm using spraying deposition (Phan et al., 2016). High spatial resolution MALDI‐MSI (Römpp & Spengler, 2013; Römpp et al., 2010; Schober et al., 2012) (down to 1.4 µm) has however been recently achievable (on single cells and tissues) but through the implementation of a sophisticated experimental workflow including an optimized pneumatic‐spray matrix application and a newly developed high‐resolution atmospheric‐pressure MALDI imaging source comprising a laser focusing objective to improve the laser focus diameter (Kompauer et al., 2017). The implementation of dry matrix applications, such as matrix sublimation, has also helped to increase the spatial resolution by providing a highly homogeneous matrix deposition with minimal lateral analyte diffusion and smaller crystal size (Gemperline et al., 2014; Hankin et al., 2007; Thomas et al., 2012). However, due to its solvent‐free nature, matrix sublimation may suffer from poor analyte extraction, decreasing the signal intensity (Phan et al., 2016). Then, when it comes to investigating the spatial distribution of small molecules (<700 Da), conventional MALDI‐MSI turns out to be challenging (Calvano et al., 2018; Kaletas et al., 2009). Indeed, upon laser irradiation, the analytes and the matrix simultaneously desorb, ionize, and potentially fragment. The ionization and fragmentation of the matrix lead to high chemical background in the low *m/z* range (Van Kampen et al., 2011), usually hampering the detection of small molecules and metabolites (<700 Da) (He et al., 2019; Lu et al., 2017). Moreover, in MALDI‐MS, the ionization of the analytes by the organic matrices is usually characterized by a low efficiency and therefore, a large excess of the organic matrices (the typical matrix/analyte ratio is 5000:1 [Chaurand, 2012]) is usually required to provide a satisfactory ionization yield of the analytes, which may in turn cause analyte‐ion suppression (Abdelhamid, 2018).

However, small molecules are of high significance in the biological field as they can play, for instance, an active role in biochemical processes such as the development of a disease or intercellular communications. Consequently, the analysis of small molecules and metabolites by MS techniques has aroused interest over the last decades. The matrix‐related problems encountered in MALDI‐MSI have thus encouraged the search for adjusted approaches. Several alternatives were proposed involving the sample preparation (such as analyte/matrix derivatization, addition of dopants, or optimized matrix application), significant instrumental improvements, and the development of novel organic matrices (Bergman et al., 2014; Calvano et al., 2018; Trim & Snel, 2016). To overcome the limitations inherent to the MALDI‐MSI technique, other LDI techniques employing solid nanosubstrates as assisting materials have also been developed over the last decades.

## 2 SALDI‐MS: AN EMERGING TECHNIQUE FOR THE ANALYSIS OF SMALL MOLECULES

### 2.1 What is SALDI?

In recent years, the emergence of a novel implementation of the LDI techniques, namely surface‐assisted laser desorption/ionization mass spectrometry (SALDI‐MS), fostered by the rapid development of nanomaterials, has created new prospects for the imaging of low molecular weight compounds (limited to 25 kDa with Pt nanosubstrates, e.g., Chiang et al., 2010), which are of particular interest, especially in the era of metabolomics and lipidomics. In SALDI‐MS, the nanosubstrates, which can be colloidal nanoparticles, solid nanostructured platforms, or sputtered metal nanoclusters, are the key elements in the desorption/ionization process, by absorbing the laser energy, enabling a rapid and sharp increase in the surface temperature leading to the analytes desorption (Chen et al., 2011; Law & Larkin 2011; Pilolli et al., 2012; Song & Cheng, 2020). Thus, while MALDI‐MS is particularly suitable for the analysis of large molecules, SALDI‐MS, which benefits from the use of nanosubstrates instead of conventional organic matrices to assist the LDI process, offers significant advantages for the analysis of small molecules by greatly limiting the interference in the low *m/z* range. In this sense, SALDI‐MS represents a complementary technique to MALDI‐MS (Phan et al., 2016; Pomastowski & Buszewski, 2019), and should not be seen as a competitive approach. The SALDI nanosubstrates have to meet the same specifications as organic matrices: they must be able to absorb the energy of the laser radiation, to promote the analytes desorption and provide a source of ionization (Chen et al., 2011; Pilolli et al., 2012).

The first example applying nanostructured inorganic matrices in “laser ionization” MS was reported as early as 1988, when Tanaka et al. used ultrafine 30‐nm cobalt nanopowders mixed with a glycerol liquid matrix as a dispersant to successfully analyze peptides and intact large proteins (up to 20 kDa) (Tanaka et al., 1988). However, it was not until 1995 that the name “SALDI‐MS” was proposed by Sunner et al. to emphasize the importance of the nanosubstrate in the laser desorption/ionization mechanism (Sunner et al., 1995).

**Figure 1:**
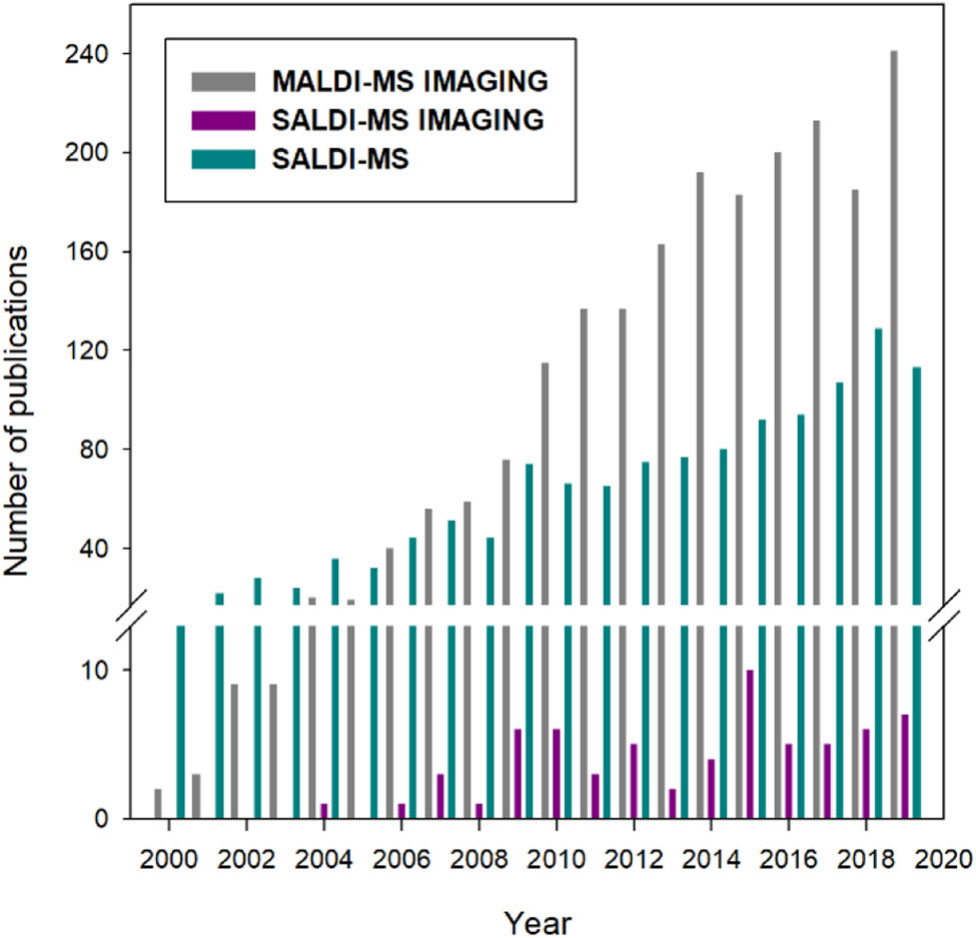
Number of publications in the field of MALDI‐MSI, SALDI‐MS, and SALDI‐MSI. Used keywords in the abstract on Scopus: “MALDI AND Imaging” for MALDI‐MSI, the sum of articles with the following keywords “SALDI,” “DIOS,” and “Nano‐PALDI,” for SALDI‐MS and the sum of articles with the following keywords “SALDI AND Imaging,” “DIOS AND Imaging,” and “Nano‐PALDI AND Imaging,” for SALDI‐MSI [Color figure can be viewed at wileyonlinelibrary.com]

However, although it has greatly evolved since these original examples, SALDI‐MS has struggled to expand and is still not extensively employed compared with the established MALDI‐MS. This is due both to fundamental (see Section 2.3) and technical reasons (see Section 3), but also probably due to some unfamiliarity with this technique. In particular, SALDI‐MS imaging (SALDI‐MSI) has only emerged in the late 2000s in the literature, 10 years later than MALDI‐MSI and is still limited to a few dozen of papers. Nevertheless, the increase in publications on SALDI‐MS over the last two decades, as shown in Figure 1, indicates a growing interest in this technique.

This surge of interest since the early 2000s is probably due as much to the boom in small molecules analyses as to the advent of nanotechnology, and in particular to the access of a wide range of nanomaterials. Indeed, many nanostructured substrates have been developed and employed for the analysis of small molecules by SALDI‐MS (Dattelbaum & Iyer, 2006; Kuzema, 2011). These nanosubstrates have found useful applications in many research areas including biomedicine (Dufresne et al., 2017; Qiao & Liu, 2010), drug analysis (Peterson, 2007), environment (Lu et al., 2017), and forensics (Guinan et al., 2015; Lim et al., 2012). For instance, SALDI‐MS has already been used to detect bone biomarkers for osteoporosis risk assessment (Pan et al., 2019), environmental pollutants from water samples (Moriwaki et al., 2018), and explosives and illicit drugs in latent fingermarks (Guinan et al., 2015; Rowell et al., 2012). However, in spite of the increased attention to SALDI‐MS, the application of this technique in an imaging context is still in a latency phase, with less than 10 publications published per year in the literature, while MALDI‐MS imaging has been booming since the early 2000s, as also shown in Figure 1.

Moreover, while there are already some reviews on SALDI‐MS, they are mainly focused on the nanosubstrates (Abdelhamid, 2019; Chu et al., 2018; Lin & Cai, 2018; Mandal et al., 2019; Muthu et al., 2018; Shi & Deng, 2016), on the SALDI mechanisms (Picca et al., 2017; Song & Cheng, 2020; Stolee et al., 2012) or on both (He et al., 2019; Iakab et al., 2019; Law & Larkin, 2011; Silina & Volmer, 2013). To our knowledge, there is no review solely dedicated to SALDI‐MS imaging, including a discussion about the nanosubstrates and their use in imaging approaches. Therefore, in this review, emphasis will be placed on SALDI‐MS in the context of imaging analyses.

### 2.2 MALDI or not MALDI? A discussion about SALDI‐MS nomenclature and acronyms

#### 2.2.1 MALDI or not MALDI?

Tanaka and his colleagues can be considered as the “SALDI pioneers” and as a result of their research, Tanaka became a co‐laureate of the Nobel Prize in Chemistry in 2002 for "the development of soft desorption ionization methods for mass spectrometric analyses of biological macromolecules.” However, in a bit of a leap, the Tanaka's work is commonly linked to the development of MALDI‐MS. We had to wait another 7 years before the "SALDI" acronym was proposed by Sunner et al. to emphasize that the use of active surfaces and structured nanomaterials as new LDI‐assisting matrices induces adaptation in the sample preparation (see Section 3) and more importantly is associated with fundamental changes in the desorption/ionization process in SALDI‐MS compared with MALDI‐MS (see Section 2.3) (Sunner et al., 1995). These reasons justify the use of distinctive acronyms for MALDI and SALDI.

#### 2.2.2 Are all acronyms necessary?

Unfortunately, since the first report of “SALDI‐MS” (in 1995) and its contemporary “DIOS” (for desorption ionization on silicon, which designates a SALDI variant triggered by porous silicon substrates) in 1999 (Wei et al., 1999), the nomenclature associated with this technique has never stopped expanding, leading to a plethora of names and acronyms often linked to the nature of the nanosubstrate, employed both in the imaging context (see Table 1) and in the general context of SALDI‐MS (see Table 2). Hence, it is already very complicated to make a comprehensive state of the art on this technique and, as the use of SALDI‐MS is expected to grow, in parallel with the fast development of improved nanosubstrates and novel LDI approaches, there is an underlying risk of facing an uncontrollable growth of new terms and acronyms in a near future that will make the understanding and reviewing of the SALDI‐MS technique increasingly difficult.

**Table 1:** Names and acronyms given to different SALDI‐MS techniques in the context of SALDI‐MS imaging with references

Acronym	Complete name	References
No acronym	MALDI‐MS with/using nanoparticles (or another nanosubstrate)	Guan et al. (2018); Jackson et al. (2014); Muller et al. (2015); Tang et al. (2011b); Zhou et al. (2017)
	Mass spectrometry using nanoparticles	Goto‐Inoue et al. (2010)
AgLDI	Silver‐assisted LDI	Baquer et al. (2020); Lauzon et al. (2015); Yang et al. (2020)
AuLDI	–	Fournelle et al. (2020)
DIOS	Desorption/ionization on silicon	Guinan et al. (2015b); Liu et al. (2007); Ronci et al. (2012); Rudd et al. (2015, 2019)
DIUTHAME	Desorption ionization using through‐hole alumina membrane	Kuwata et al. (2020)
GALDI	Graphite‐assisted LDI	Cha and Yeung (2007); Zhang et al. (2007)
LDI	Laser desorption/ionization	Dupré et al. (2012); Dufresne et al. (2013, 2016); Hansen et al. (2019); Jun et al. (2010); McLaughlin et al. (2020); Misiorek et al. (2017); Niziol et al. (2019); Niziol and Ruman (2013b); Rafols et al. (2018); Schnapp et al. (2016); Sekula et al. (2015a); Tseng et al. (2017)
NALDI	Nano‐assisted LDI	Creran et al. (2012); Ronci et al. (2012); Tata et al. (2012, 2014); Vidova et al. (2010)
	Nanomaterial‐assisted LDI	Kim et al. (2011); Qiao and Liu (2010)
	Nanoparticle‐assisted LDI	Huang et al. (2015)
	Nanostructure‐assisted LDI	Krasny et al. (2015); Skriba and Havlicek (2018)
Nano‐PALDI	NanoParticle‐assisted LDI	Ageta et al. (2009); Hayasaka et al. (2010); Shiono and Taira 2020); Shrivas et al. (2011); Taira et al. (2008)
NAPA‐LDI	NanoPost array LDI	Fincher et al. (2019a, 2019b, 2020a, 2020b); Samarah and Vertes (2020); Stopka and Vertes (2020); Stopka et al. (2016)
nPALDI	nanoParticle‐assisted LDI	Morosi et al. (2013)
NIMS	Nanostructure‐initiator MS	Calavia et al. (2012); Greving et al. (2011); Lee et al. (2012); O'Brien et al. (2013); Palermo (2020); Palermo et al. (2018); Patti et al. (2010a, 2010b); Yanes et al. (2009)
	Nanostructure imaging MS	Guinan et al. (2015); Palermo et al. (2018)
ME‐NIMS	Matrix‐enhanced nanostructure initiator MS	Moening et al. (2016)
ME‐SALDI	Matrix‐enhanced surface‐assisted LDI	Brown et al. (2015); Liu and He (2009); Liu et al. (2009)
MILDI	Matrix implantation LDI	Muller et al. (2017)
Pt‐SALDI	Surface‐assisted laser desorption/ionization with sputter‐deposited platinum film	Ozawa et al. (2016)
SALDI	Surface‐assisted LDI	Chau et al. (2017); Cheng et al. (2016); Dutkiewicz et al. (2019); Iakab et al. (2020); Lopez de Laorden et al. (2015); Müller et al. (2020); Niziol et al. (2016); Phan et al. (2016); Wang et al. (2020a, 2020b)
SPILDI	Silica plate imprinting LDI	De Oliveira et al. (2014)
SP‐LDI	Silica plate LDI	Ferreira et al. (2014)

**Table 2:** Some other names of SALDI‐MS (not met in the imaging context) with references

Acronym	Complete name	References
No acronym	Inorganic material‐assisted LDI	Qiao and Liu (2010)
	Matrix‐less mass spectrometry	Niziol and Ruman (2013a)
	Matrix‐free LDI	Niziol et al. (2013)
CALDI	Cation‐assisted LDI	Ha et al. (2008)
DIOM	Desorption/ionization on mesoporous silicate	Chang‐soo Lee et al. (2007)
GALDI	Graphene‐assisted LDI	Abdelhamid and Wu (2012)
	Graphene oxide assisted LDI	Abdelhamid and Wu (2015)
	Gold‐assisted LDI	Abdelhamid and Wu (2016)
	Geomatrix‐assisted LDI	Yan et al. (2007)
NALDI	Nanowire‐assisted LDI	Kang et al. (2005)
	Nanostructure‐assisted LDI	Wyatt et al. (2010)
NPs‐ALDI	NanoParticle‐assisted LDI	Abdelhamid (2018)
MELDI	Material‐enhanced LDI	Feuerstein et al. (2006); Rainer et al. (2011)
mf‐MELDI	matrix‐free material‐enhanced LDI	Qureshi et al. (2014)
MILDI	Matrix‐implanted LDI	Novikov et al. (2004); Tempez et al. (2005)
SPALDI	Silicon nanoParticle assisted LDI	Wen et al. (2007)
	Silicon nanoPowder assisted LDI	Dagan et al. (2006)

Furthermore, the use of some acronyms is not always appropriate. As a matter of fact, some acronyms refer to different techniques and do not have the same meaning, such as NALDI or GALDI. Some other techniques are referred to by several acronyms, such as nanoparticle‐assisted laser desorption/ionization. Again, the understanding of the literature could be affected by this ambiguity in terminology. This messy nomenclature is concerning and there is an urgent need to clarify the terminology and unify the field concepts and theories.

Certainly, some differences lie between all the SALDI sub‐categories such as the physicochemical properties of the nanosubstrate (linked to its nature and structure), the way in which the nanosubstrate is employed, or whether or not other LDI assisting molecules are added to the sample. However, we believe that the differences between the above techniques are not sufficiently significant to warrant the creation and use of new terminologies. Therefore, we suggest the use of a generic appellation for all these techniques. In this context, common sense would dictate recommending the most widely and frequently accepted terms in the literature. In this context, the terms DIOS and SALDI seem to be the most employed. In addition, the IUPAC's recommendations for the Definitions of Terms Relating to Mass Spectrometry (2013) (Murray et al., 2013) also support the use of DIOS and SALDI. The definitions provided by the IUPAC are:

**DIOS** (desorption ionization on silicon): Soft ionization alternative to matrix‐assisted desorption/ionization involving laser desorption ionization of a sample deposited on a porous silicon surface.

**SALDI** (surface‐assisted laser desorption/ionization): Class of matrix‐free laser desorption ionization techniques for biological macromolecules. Note: an example is desorption ionization on silicon (DIOS).

Nevertheless, the terms DIOS and SALDI seem to be redundant since DIOS is a particular case of SALDI, employing porous silicon as substrate. Thus, we would suggest the use of the term “SALDI” as a general designation as (i) the term encompasses a very large number of sub‐techniques and (ii) it reminds us that the technique belongs to the wider group of LDI techniques.

#### 2.2.3 Is the IUPAC definition of SALDI outdated?

Other issues now concern the SALDI definition proposed by the IUPAC, which no longer corresponds to the technique in the field of MS. First, there is some controversy over the so‐called “matrix‐free” methods, since there is in fact a matrix involved in SALDI. The unique difference is that these matrices are simply not conventional organic matrices as designed in MALDI‐MS but rather nanostructured substrates used to assist the LDI process. Second, SALDI‐MS has been employed to analyze a wide variety of molecules, not just biological macromolecules. Third, SALDI is more commonly used for the analysis of small molecules rather than macromolecules. We would therefore suggest to adapt the IUPAC's definition of SALDI, following for example the criteria already proposed by Law and Larkin in 2011 (Law & Larkin, 2011) as well as recently exposed criteria, including the enhanced specificity and sensitivity of this technique (compared with MALDI‐MS) due to the high affinity of the nanosubstrate with specific analytes (see Section 3.3).

### 2.3 SALDI‐MS fundamental mechanisms

The understanding of the fundamental mechanisms underlying the LDI processes remains the topic of a lively discussion in the scientific community. While the mechanistic aspects of MALDI have gained sizeable knowledge over the past decades (see Dreisewerd, 2003; Jaskolla & Karas, 2011; Karas & Krüger, 2003; Knochenmuss, 2006; Knochenmuss & Zenobi, 2003; Lee et al., 2019; Niehaus & Soltwisch, 2018; Zenobi & Knochenmuss, 1998, for example), the study of the key principles of SALDI is still in a nascent state (Cheng & Ng, 2020; Law & Larkin, 2011; Picca et al., 2017; Song & Cheng, 2020; Stolee et al., 2012; Vertes, 2007) and represents one of the most controversial part of the SALDI research (Law & Larkin, 2011), hindering its development and applications (Zhu et al., 2020). The elucidation of the SALDI mechanistic aspects is far from easy because many factors affect the analytical performance of the SALDI processes and the proper impact of each factor remains ambiguous (Picca et al., 2017). Some of these factors are related to the SALDI nanosubstrate, such as the surface morphology (Zhu et al., 2020) (e.g., shape, size, and porous nanostructure) and nature, which define its physicochemical properties (e.g., photoabsorption efficiency, thermal conductivity, melting point) (Lai et al., 2016). Other parameters depend on the nature of the analytes including their chemical properties and the interactions between the nanosubstrate and the analytes (Picca et al., 2017). Finally, some parameters fall into experimental operating parameters, such as the excitation laser irradiation parameters (e.g., irradiance, wavelength, number, length, energy, and frequency of the pulses) and ionization mode (positive or negative) (Picca et al., 2017). The only point on which the scientific community comes to some sort of agreement is that the nanosubstrates play a major role in the desorption/ionization mechanisms, by absorbing the laser energy, enabling a rapid and sharp increase in the surface temperature, and that both thermal and non‐thermal processes may be involved in the overall SALDI‐MS process (Law & Larkin, 2011; Song & Cheng, 2020).

Different models have attempted to explain (at least partially) the SALDI mechanisms. These models have generally focused on one of the two distinct, but concomitant, contributions of the SALDI process: the desorption or the ionization. On the one hand, desorption is thought to mainly occur via thermal processes including the rapid and highly localized heating of the nanosubstrate (see Section 2.3.1), even if some other non‐thermal processes may also help the analyte desorption, such as surface restructuring or destruction (see Section 2.3.2). On the other hand, ionization has been presented as a non‐thermal process (see Section 2.3.2) but remains misunderstood as it can be promoted by various phenomena, including charge transfers, photo‐ionization reactions, or surface melting/destruction.

This section summarizes the main hypotheses proposed to explain the SALDI fundamental mechanisms. It is however impossible to take all the fine details presented in the literature into account and this section will be restrained to a simplified explanation of the SALDI mechanistic aspects. Interested readers are therefore invited to consult the various references mentioned throughout this section.

#### 2.3.1 Thermal processes promoting analyte desorption

The SALDI desorption process has been widely recognized as a laser‐induced thermal‐driven phenomenon (Lai et al., 2016; Ng et al., 2015; Picca et al., 2017). This mechanism is based on the rapid heating of the nanosubstrate, coupled with heat confinement effects (Picca et al., 2017), resulting from the interaction of a nanosecond‐pulsed laser with the nanostructure (Song & Cheng, 2020). Thus, upon laser irradiation, the local temperature around the nanosubstrate can be very high, high enough to desorb most kinds of analytes (Ng et al., 2015). Nanosubstrates, characterized by a strong absorbance in the UV‐Vis region, a low heat capacity, and a reduced thermal conductivity (related to their size, surface roughness, and electron thermal conductivity [Picca et al., 2017]), might therefore play an active role in this mechanism (Song & Cheng, 2020). The local thermal density has to be high and it is essential that the nanosubstrate has a low thermal conductivity so that the “thermal spike” (Vineyard, 1976) does not dissipate too rapidly. Hence, an efficient energy transfer from the nanosubstrate to the analytes seems to induce efficient desorption (Song & Cheng, 2020) and signal enhancement in SALDI‐MS.

#### 2.3.2 Non‐thermal processes behind analyte desorption and ionization

Non‐thermal processes are also generally proposed as possible mechanisms for desorption and ionization in SALDI‐MS. On the one hand, non‐thermal processes such as laser‐induced surface restructuring or destruction are mentioned as possible mechanisms for the desorption process in SALDI‐MS (Song & Cheng, 2020). On the other hand, the ionization in SALDI may also be driven by different non‐thermal processes. However, the ionization mechanism remains largely unclear as various pathways can promote ionization, such as the emission of hot electrons, the presence of pre‐existing ions in the sample, photoionization reactions between the solvent molecules (trapped in the nanostructure) and the analytes, and proton or electron transfer between the surface and the analytes (Luo et al., 2005). The surface melting and destruction have also been suggested as potential parts of the SALDI ionization mechanisms. Indeed, ions and charged clusters originating from the substrates are often detected during a SALDI‐MS experiment (Song & Cheng, 2020).

**Figure 2:**
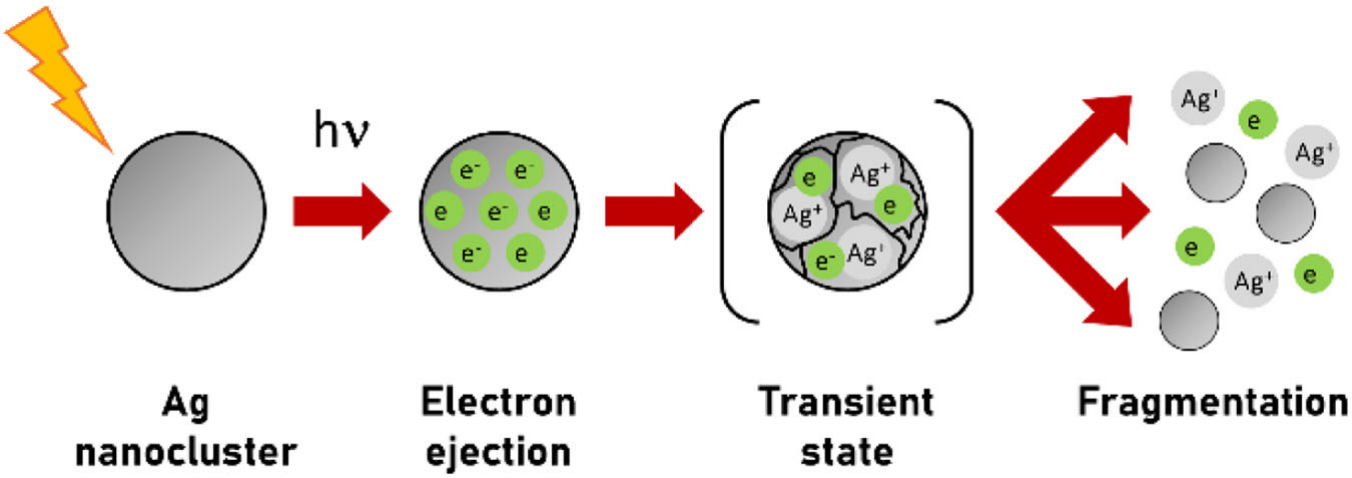
Fragmentation of silver nanoparticle upon laser irradiation. The transient aggregate formed via the photoejection of electrons is considered as a precursor for complete fragmentation of the nanoparticle. Adapted with permission from Kamat et al. (1998).© 1998 American Chemical Society [Color figure can be viewed at wileyonlinelibrary.com]

In particular, plasmonic nanomaterials (such as gold‐, silver‐, and platinum‐based nanoparticles) exhibit a high photochemical activity when they are irradiated by a UV‐Vis laser (Kamat et al., 1998), facilitating the conversion of light energy into chemical energy by generating high‐energy electrons (called hot electrons [Li et al., 2018]) and paired with holes (Cheng & Ng, 2020). The ionization process in SALDI‐MS has been mainly considered as the result of the ejection of hot electrons, which are the most likely source of initial charges in SALDI, and their subsequent transfer from the nanostructure to the adjacent molecules (Li et al., 2018). Indeed, upon nanosecond‐pulsed laser excitation, noble metal nanoclusters can become positively multiply‐charged due to the release of a high quantity of electrons (Shoji et al., 2008). The nanoparticle then carries so many charges that it eventually become unstable, because the Coulomb repulsive forces between these charges exceed the cohesive forces operating inside the nanocluster. Coulomb explosion finally leads to the spontaneous fragmentation of the nanoparticle (Shoji et al., 2008), as shown in Figure 2, resulting in the ejection of quite a number of electrons (Werner & Hashimoto, 2011) and nanosubstrate ions.

However, the hot electrons are only one side of the coin. Indeed, recently, Cheng and Ng brought the “Hidden Heroes,” namely the holes generated simultaneously with the hot electrons, out of the shadows (Cheng & Ng, 2020). They emphasized the importance of the contributions of the holes, usually disregarded, in a new “charge‐driven” desorption mechanism. This mechanism involves positive hole‐containing nanosubstrates formed via the hot‐electron transfer from the substrate to the conductive support (e.g., MALDI plate adapter). The holes reduce the interactions between the analyte ions and the nanosubstrate surface and achieve Coulomb repulsion between the positively charged nanosubstrate and the analyte ions, allowing their desorption in positive ionization mode.

**Figure 3:**
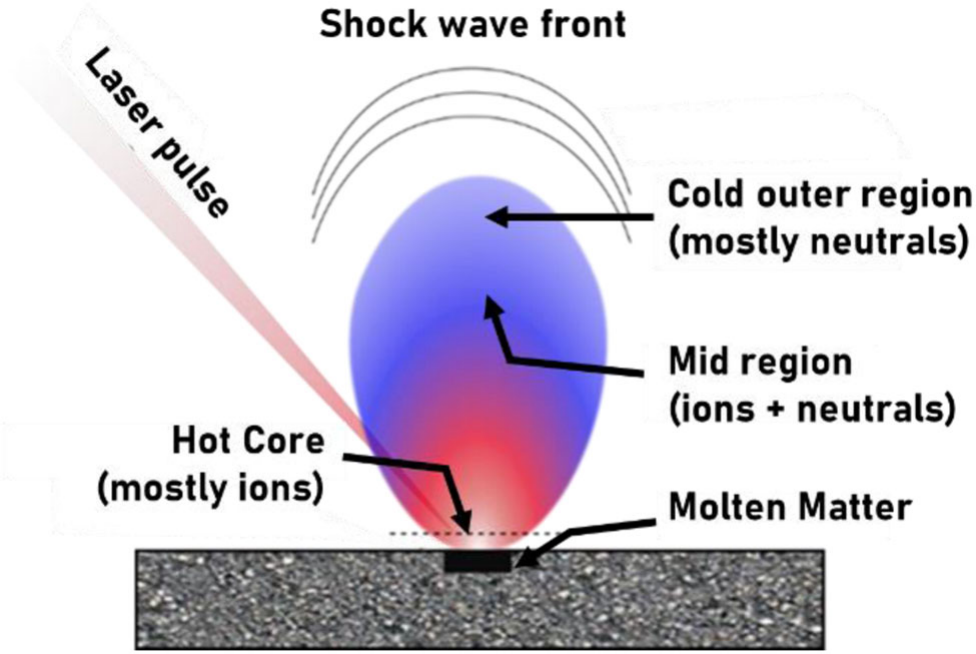
Schematic illustration of laser‐induced plasma. Adapted from Chaudhary et al. (2016) [Color figure can be viewed at wileyonlinelibrary.com]

In another approach, an analogy is made between SALDI ionization and laser ablation. Indeed, the surface melting/destruction occurring in SALDI through laser irradiation can be seen as a similar process to the laser ablation process if enough laser energy is absorbed by the nanosubstrate (Song & Cheng, 2020). Without going into details not covered in this review (more information can be found in the work of [Song & Cheng, 2020]), laser ablation is a non‐thermal process generating a plasma, induced by laser irradiation of the sample surface, as shown in Figure 3. Surface melting, dissociation, vaporization, ionization, and removal by the shock wave are all parts of the laser ablation process (Song & Cheng, 2020). As shown in Figure 3, the laser‐induced plasma contains a variety of species such as electrons, neutrals, excited neutrals and ions. Numerous gas‐phase collisions can occur among species inside the laser‐induced plasma due to the dense population of various energetic species. SALDI‐MS ionization process may therefore result from these collisions, which can also produce ions from analytes in the gas phase (Song & Cheng, 2020).

#### 2.3.3 Exploring SALDI‐MS processes with “thermometer ions”

The correlation between internal energy transfer and ion desorption efficiency is generally probed to explore the LDI processes (Picca et al., 2017). Internal energy transfer investigations have already contributed to the (partial) understanding of MALDI and ESI ionization sources and are expected to shed light on the fundamental mechanisms of SALDI.

**Figure 4:**
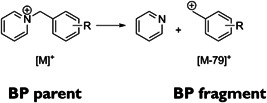
General fragmentation scheme of the benzylpyridinium ions

In this context, as originally proposed by De Pauw et al., preionised substituted benzylpyridinium (R·BP^+^) salts can be used as chemical thermometers probing the extent of heat transfer from the nanosubstrate to the (R·BP^+^) ions during the LDI process (Collette & De Pauw, 1998). Laser desorbed (R·BP^+^) ions, possessing a greater amount of internal energy than the critical energy of the unimolecular dissociation energy (E_0_), could undergo a simple cleavage of the C—N bond between the benzyl C and the pyridine N, producing (R·BP—Pyridine)^+^ “fragment ions” (Figure 4). The extent of fragmentation can be evaluated from the relative proportion of the survived intact (R·BP^+^) “parent ions” to the total intensity of desorbed benzylpyridinium ions, expressed by the survival yield (SY), defined as

$$\mathrm{SY}=\frac{I_{P}}{I_{P}+I_{F}},$$

where *I*_P_ and I_F_ indicate the intensity of the (R·BP^+^) parent ions and (R·BP—Pyridine)^+^ fragment ions, respectively. Several SY methods can be employed (namely the “original” (Collette & De Pauw, 1998; Derwa et al., 1991; Tang et al., 2009), the “modified” (Tang et al., 2009) and the “simplified” (Bian & Olesik, 2020; Luo et al., 2002; Tang et al., 2009) SY methods) to evaluate the extent of internal energy transfer during the laser‐induced desorption process. A comprehensive explanation of the SY procedure is again beyond the scope of this review but interested readers may consult the publications of (Greisch et al., 2003) and (Gabelica & De Pauw, 2005), for example, in addition to the references already mentioned in this paragraph.

**Figure 5:**
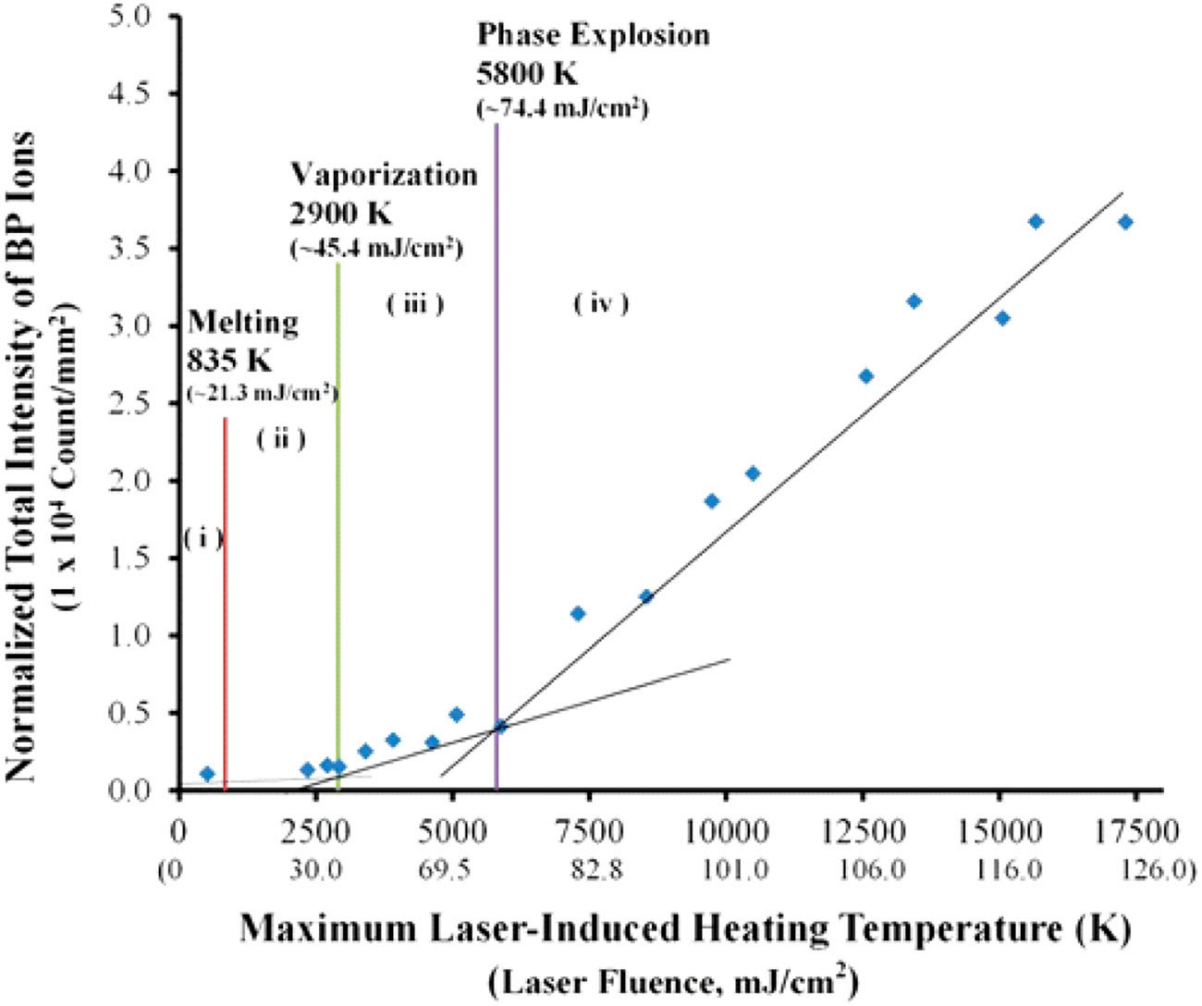
Effect of the computed maximum laser‐induced heating temperature of the AuNP on the normalized total intensity of BP ions desorbed from AuNPs. (i) Solid, (ii) liquid, (iii) gas, and (iv) phase explosion regions are also labeled. Reprinted with permission from Lai et al. (2016). © 2016 American Chemical Society [Color figure can be viewed at wileyonlinelibrary.com]

In particular, some SY procedures and other experiments using benzylpyridinium thermometer ions have brought the foundation stones for the elucidation of the complex SALDI‐MS fundamental processes. For instance, Lai et al. investigated the effect of the different phase transition steps of gold nanoparticles on benzylpyridinium ions desorption efficiency upon laser irradiation (Lai et al., 2016). They plotted the experimental total intensity of desorbed (R·BP^+^) ions as a function of theoretically calculated maximum laser‐induced heating temperatures of the gold nanoparticles, as shown in Figure 5. They proposed that when the gold nanoparticles remain solid (i) or liquid (ii), the (R·BP^+^) desorption is mainly driven by the thermal desorption process, and the total intensity of desorbed (R·BP^+^) ions remains low (Lai et al., 2016). This is confirmed by the calculations of Pyatenko et al. which concluded that the photothermal mechanism prevails at low laser intensities (Pyatenko et al., 2009). In contrast, when the laser energy is high enough to vaporize the nanoparticles, the total intensity of desorbed (R·BP^+^) ions increases steadily (iii) (Lai et al., 2016). Finally, when laser energy exceeds a threshold value through region (iv), the phase explosion of the nanosubstrate occurs, leading to a significant increase of the total intensity of desorbed (R·BP^+^) ions (Lai et al., 2016).

**Figure 6:**
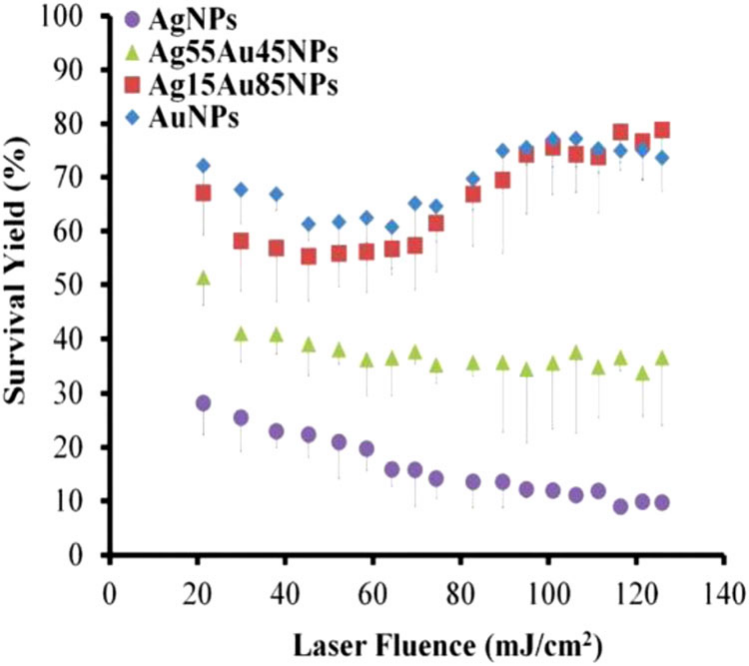
Effect of laser fluence on the survival yield of BP ions desorbed from AgNPs, Ag55Au45NPs, Ag15Au85NPs and AuNPs. Reprinted with permission from Lai et al. (2017). © 2017 PCCP Owner Societies [Color figure can be viewed at wileyonlinelibrary.com]

Moreover, the same research group studied the influence of the tuning of the metal contents of Ag‐Au alloy nanoparticles on the SALDI desorption efficiency and heat transfer (Lai et al., 2017). They found that the composition of the nanosubstrate affects the ion desorption efficiency but also the extent of heat transfer from the substrate to the analyte, as shown in Figure 6.

Overall, these fundamental studies indicate that the SALDI processes are a combination of both thermally and non‐thermally energy transfers depending on the nature of the substrate and the energy brought to the sample by the exciting laser.

## 3 ANALYTICAL STRATEGIES FOR SALDI‐MS IMAGING

As for every MS experiments, the quality of the sample is paramount (Chaurand, 2012), but sample preparation is also one of the crucial steps, determining the success of the imaging analysis (Goodwin, 2012; Phan et al., 2016). A variety of sample preparation procedures have been developed to fulfill all requirements of each imaging technique. All steps of the preparation will often influence the results and therefore have to be optimized, from sample collection to surface treatment prior mass analysis (Amstalden van Hove et al., 2010). In particular, care must be taken to preserve the integrity and the spatial distribution of the analytes in the sample, which is critical in imaging analyses (Fournelle et al., 2020).

**Figure 7:**
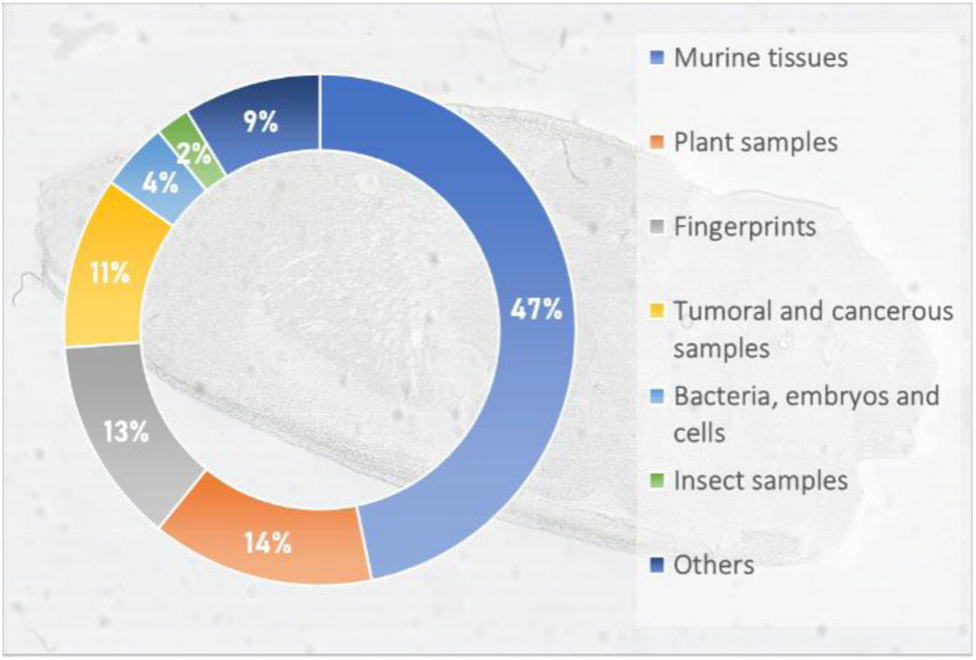
Sample types in SALDI‐MSI [Color figure can be viewed at wileyonlinelibrary.com]

While countless samples have already been studied by MALDI‐MSI in disciplines as varied as pharmaceutical research (Schulz et al., 2019; Swales et al., 2019), ecotoxicology (Lagarrigue et al., 2016), plant biology (Boughton et al., 2016), biomedicine (Schwamborn et al., 2017), and molecular biology (Cornett et al., 2007), the variety of samples that have already been imaged by SALDI‐MSI remains rather limited, as shown in Figure 7 and Table 3, 4. Among these samples, the majority of reported SALDI‐MS imaging analyses focused on murine tissue sections (about 50% of which were mouse brain sections). This is not surprising as the most imaged tissue type, regardless of the MSI technique, is mouse brain due to its small size, its characteristic internal structure (which is nowadays well documented), and its ease of sectioning (Chughtai & Heeren, 2010).

**Table 3:** Summary of the different types of samples analyzed by SALDI‐MSI

Sample type	References
Murine brain	Ageta et al. (2009); Baquer et al. (2020); Cha and Yeung (2007); Dufresne et al. (2013); Fincher et al. (2019a, 2020b); Goto‐Inoue et al. (2010); Guan et al. (2018); Iakab et al. (2020); Kim et al. (2011); Lee et al. (2012); Lopez de Laorden et al. (2015); Muller et al. (2017); Müller et al. (2020); Patti et al. (2010a, 2010b); Rafols et al. (2018); Shrivas et al. (2011); Stopka et al. (2016); Taira et al. (2008); Wu et al. (2017); Yanes et al. (2009); Yang et al. (2020); Zhou et al. (2017)
Murine kidney	Baquer et al. (2020); Chau et al. (2017); Cheng et al. (2019a, 2019b); Dufresne et al. (2013); Iakab et al. (2020); Krasny et al. (2015); Stopka et al. (2016) Tata et al. (2014); Vidova et al. (2010)
Murine lung	Fincher et al. (2020a, 2020b)
Murine colon	Palermo et al. (2018)
Murine pancreas	Baquer et al. (2020)
Murine liver	Dufresne et al. (2013, 2016); Iakab et al. (2020); Liu et al. (2007)
Murine retina	Hayasaka et al. (2010)
Murine heart	Jackson et al. (2014)
Murine testis	Dufresne et al. (2013)
Fingerprints	Cheng et al. (2016); Guinan et al. (2015a, 2015c); Iakab et al. (2020); Lauzon et al. (2015); Niziol and Ruman (2013b); Sekula et al. (2015a, 2015b); Schnapp et al. (2016); Skriba and Havlicek (2018); Tang et al. (2010); Yang et al. (2019); Wang et al. (2020b)
Tumoral and cancerous samples	Huang et al. 2015; Morosi et al. (2013); Niziol et al. (2016, 2020); O'Brien et al. (2013); Rudd et al. (2019); Tang et al. (2011b); Tata et al. (2012); Tseng et al. (2017); Zhou et al. (2017)
Flowers	Dutkiewicz et al. (2019); Jun et al. (2010); Niziol and Ruman (2013b); Patti et al. (2010b); Wang et al. (2020)
Leaves	Ozawa et al. (2016)
Fruits	De Oliveira et al. (2014); Niziol et al. (2019); Zhang et al. (2007)
Roots and bulbs	Hansen et al. (2019); Jun et al. (2010); Misiorek et al. (2017); Sekula et al. (2015a); Shiono and Taira (2020)
Stems	Dutkiewicz et al. (2019); Niziol and Ruman (2013b); Patti et al. (2010b)
Seeds	Hansen et al. (2019)
Bacteria and fungi	Chen et al. (2018); Dutkiewicz et al. (2019)
Cells and embryos	Ferreira et al. (2014); Liu et al. (2007); McLaughlin et al. (2020); Stopka and Vertes (2020)
Insect samples	Phan et al. (2016); Schnapp et al. (2016)
Banknotes and documents	Tang et al. (2011a)
Peptide droplets	Dupré et al. (2012)
Rabbit adrenal gland	Dufresne et al. (2016)
Human skin tissues	Fincher et al. (2019b, 2020a)
Marine mollusc gland	Ronci et al. (2012); Rudd et al. (2015)

**Table 4:** Summary of the deposition/utilization of the nanosubstrates

	Spraying	Imprinting	Deposition	Sputtering	Implantation
Instrumentation	Easy‐to‐handle instrumentation, automated devices allow a fairly good control of the spraying parameters	Does not require any particular instrumentation	Does not require any particular instrumentation	Requires sophisticated instrumentation and the precise control of the sputtering parameters	Requires specialized instrumentation for NP implantation
Spatial resolution	Limited by the migration of the analytes due to the solvent and by the aggregation and diffusion of the nanosubstrates. Usual spatial resolution varies between 10 and 200 µm	Limited by the smudging of the spatial details during the imprinting step. Spatial resolution varies between 50 and 200 µm, 150 µm is usually employed	Only limited by the instrumentation (i.e., laser spot size and moving stage). Currently a spatial resolution down to 10 µm can be achieved	Only limited by the instrumentation (i.e., laser spot size and moving stage). Currently a spatial resolution down to 10 µm can be achieved. Dufresne et al. also employed a 5‐µm resolution (Dufresne et al., 2013)	The dryness of the method and the lack of turbulent flow avoid any physical movement of the analytes and thus allow a high spatial resolution. A 50‐µm resolution is usually employed but perhaps needs optimization.
Other advantages	Large selection of commercially available colloids	Easy and rapid procedure	Easy and rapid procedure	Eliminates nanoparticle aggregation, high reproducibility, renders surfaces conductive (allows imaging of samples on nonconductive surfaces)	Eliminates nanoparticle aggregation, high reproducibility of the implantation
Other limitations	Often requires stabilizing agents, which may cause interference in the low m/z range and/or ion suppression of the analytes	Lack of sensitivity for low‐abundant species, inefficiency in transferring some analytes	Need for very thin tissue sections (<5 µm), thus usually requiring embedding of the sample. Thicker sections affect conductivity and lead to low ionization efficiency, may suffer from limited sensitivity. Imaging artefacts may result from the different behaviors of histologically different regions of the tissue section upon laser irradiation	High purity metals and argon are expensive	High purity metals and argon are expensive
Some examples of nanosubstrates	AuNPs (Goto‐Inoue et al., 2010; McLaughlin et al., 2020; Müller et al.^2020^; Phan et al., 2016), AgNPs (Guan et al., 2018; Hayasaka et al., 2010; Jun et al., 2010), Graphene oxide (Zhou et al., 2017), TiO_2_NPs (Morosi et al., 2013; Shrivas et al., 2011; Yang et al. 2019), Graphite (Cha & Yeung, 2007; Zhang et al., 2007)	AuNPET (Misiorek et al., 2017; Niziol et al., 2016; Sekula et al., 2015b), ^109^AgNPET (Niziol & Ruman, 2013a; Niziol et al., 2019, 2020) Etched Ag foils (Schnapp et al., 2016), TiO_2_nanowire surface (Dutkiewicz et al., 2019), Porous silicon (Ronci et al., 2012; Rudd et al., 2015), Commercial NALDI target (Krasny et al., 2015; Skriba & Havlicek, 2018; Tata et al. 2012, 2014; Vidova et al. 2010), Silica plate (De Oliveira et al., 2014), AuBSi (Iakab et al., 2020)	Silicon nanopost array (Fincher et al., 2019a, 2019b, 2020a, 2020b; Stopka et al., 2016), Silica plate (Ferreira et al., 2014), (Modified) etched silicon (Guinan et al., 2015c; Liu et al., 2007; O'Brien et al., 2013; Patti et al. 2010b; Yanes et al., 2009), SiO_2_NPs (Dupré et al., 2012)	Pure metal: Au (Chau et al., 2017; Dufresne et al., 2016; Hansen et al., 2019; Rafols et al. 2018; Tang et al., 2010, 2011a, 2011b), Ag (Baquer et al., 2020; Dufresne et al., 2013; Hansen et al., 2019; Lauzon et al., 2015; Yang et al., 2020), Pt (Hansen et al., 2019; Ozawa et al., 2016), Cu (Hansen et al., 2019), Ti (Hansen et al., 2019), Ni (Hansen et al., 2019); Pure metal oxide: ITO (Lopez de Laorden et al., 2015)	Pure metal: Ag (Jackson et al., 2014; Muller et al. 2015, 2017)

Obviously, the sample preparation depends on the sample itself. Each sample needs its own preparation optimization, which is impracticable to cover in this review. Therefore, only the most common sample preparation protocols in the frame of SALDI‐MS imaging will be discussed.

**Figure 8:**
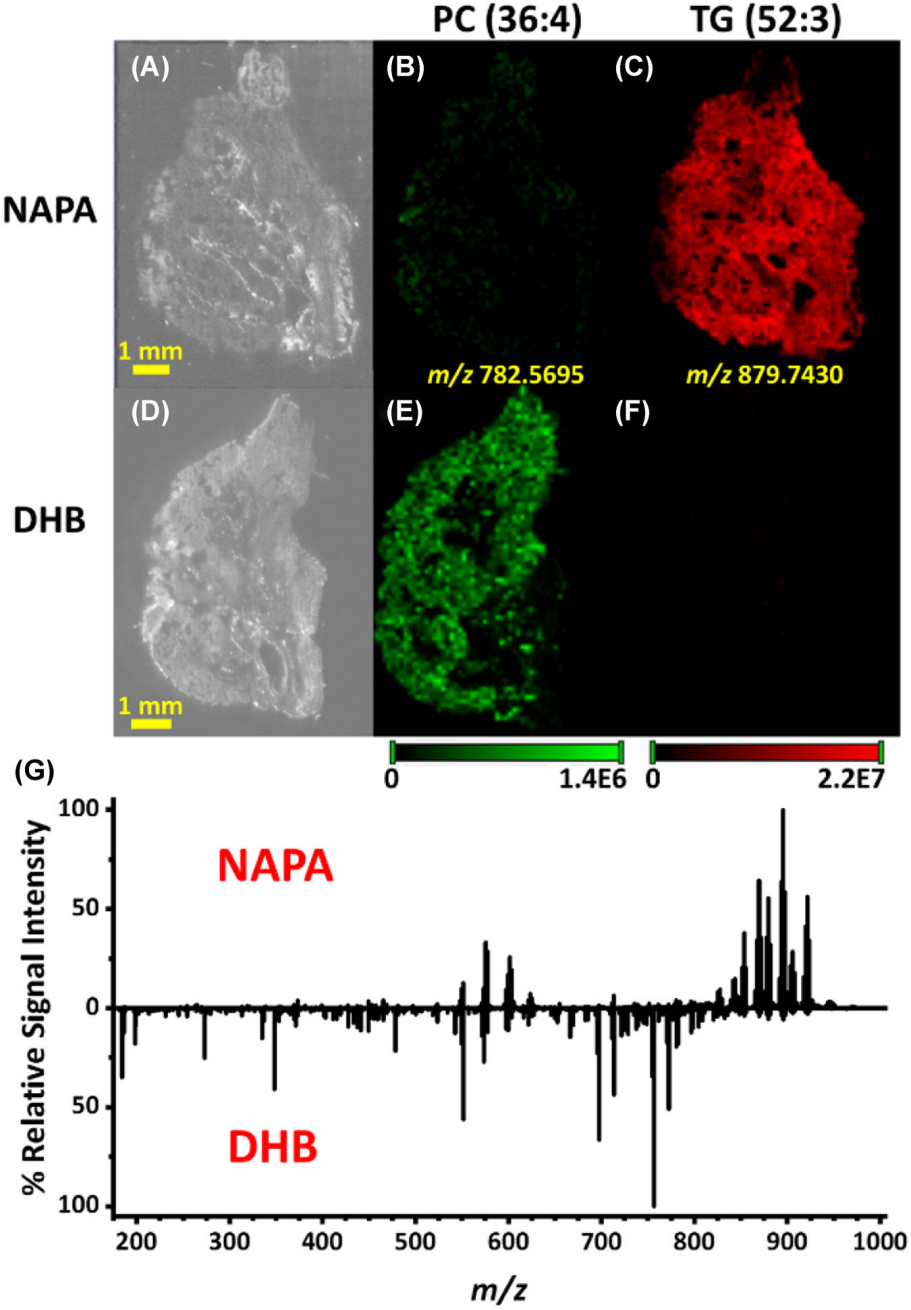
(A and D) Optical images of serial mouse lung tissues sections. (B and E) Distribution of the [M+H]^+^ ionic species of a phospholipid (PC(36:4)) acquired by NAPA‐LDI‐MSI (B) and MALDI‐MSI (E). (C and F) Distribution of the [M+Na]^+^ adduct of a triglyceride (TG(52:3)) acquired by NAPA‐LDI‐MSI (C) and MALDI‐MSI (F). (G) Comparison of the averaged mass spectra of the entire tissue region acquired by NAPA‐LDI‐MSI (NAPA) and MALDI‐MSI (DHB). Reprinted with permission from Fincher et al. (2020a). © 2019 John Wiley &amp; Sons, Ltd. [Color figure can be viewed at wileyonlinelibrary.com]

Overall, the analytical strategy implemented in SALDI‐MSI is very similar to the MALDI‐MSI analytical workflow. Thus, SALDI‐MSI experiments do not require any significant change in instruments and protocols compared with MALDI‐MSI, making this technique accessible to all laboratories familiar with MALDI‐MSI. Moreover, as SALDI and MALDI MSI are complementary techniques, their similar instrumentation is a real asset in the context of multimodal MSI. Their combination has already proved to allow a better molecular coverage. For example, Fincher and coworkers took profit of this complementarity to image lipids in biological samples. The analysis of neutral lipids (e.g., triglycerides [TG] and hexosylceramides) remains challenging by MALDI‐MSI due to ion suppression by phospholipids. In Fincher's studies, silicon nanosubstrates (NAPA) were able to selectively ionize neutral lipids (Fincher et al., 2020a, 2020b). In contrast, MALDI‐MSI provided higher signals for phosphatidylcholines (PC, a class of phospholipids) compared with SALDI‐MSI (Fincher et al., 2020a) (Figure 8).

However, despite the similarities between SALDI and MALDI experimental workflows, some characteristics are specific to SALDI‐MSI such as the influence of the nanosubstrate chemical composition and shape (Section 3.3) on the LDI mechanisms (see Section 2.3), the possibilities of nanosubstrate functionalization for targeted SALDI‐MSI (see Section 3.4) or some nanosubstrate deposition methods specific to SALDI‐MSI (see Section 3.5).

In this section, to provide the reader with a complete overview of the SALDI‐MSI analysis, each part of the SALDI‐MSI sample preparation will be discussed, even those already commonly encountered in MALDI‐MSI.

### 3.1 Stabilization of the sample

After sample collection, the sample degradation and analyte delocalization have to be avoided. In this respect, the embedding in a protective material and/or flash‐freezing helps to preserve the sample integrity. In general, most samples used for MSI analyses are fresh‐frozen and chemically unmodified (Chughtai & Heeren, 2010). Indeed, other treatments, such as formalin fixation, might not be compatible with MS analyses due to the formation of chemical cross‐links in the sample and/or interfering signals in the *m/z* range of interest (Buchberger et al., 2018; Kaletas et al., 2009). This is also the case for SALDI‐MSI experiments, in which the largest part of the analyzed samples are either fresh‐frozen (~40% of the samples reported in Table 3) or non‐stabilized samples (i.e., without any treatment or freezing) (~30% of the samples reported in Table 3) mainly encountered in the “imprinting” and “deposition” sample preparation (see Section 3.5).

Fresh‐freezing is, for instance, performed using an isopentane bath chilled with liquid nitrogen or dry ice (Goodwin, 2012). This procedure allows to rapidly freeze samples that contain high amount of water so that the water does not have time to crystallize, maintaining water in a vitreous form that does not expand under solidification. The formation of ice crystals in the sample often induces physical distortion in the samples as well as tissue cracking. Other freezing procedures employ liquid nitrogen or dry ice. However, the use of liquid nitrogen has to be avoided as a vapor barrier forms at the interface of the sample with the liquid nitrogen, which boils when it gets in contact with the sample to be frozen. This lowers the cooling rate and leads to unpredictable freezing process, which in turn inflicts damage to the tissue. However, obtaining fresh‐frozen samples without embedding is sometimes difficult, especially in the medical field in which the samples are routinely alcohol‐ or formalin‐fixed and paraffin‐embedded (FFPE) just after biopsy. In that case, a paraffin removal step using a xylene wash will have to be carried out prior imaging as paraffin suppresses ionization (Ly et al., 2016). However, the deparaffinisation steps using xylene can alter the distributions of molecular species soluble in organic solvents (such as lipids) or even wash out these compounds from the sample (Pietrowska et al., 2016). Samples can also be embedded in other materials, such as optimal cutting temperature (OCT) polymeric matrices, epoxy resin, carboxymethyl cellulose or gelatin. Embedded samples are generally easier to be cut in sections than fresh‐frozen samples (Chughtai & Heeren, 2010). However, the flip side of the embedding step is that the protective material ionizes as well. OCT matrices, for instance, ionize easily, which leads to significant spectral interference and ion suppression in the low *m/z* range (Phan et al., 2016; Schwartz et al., 2003). Despite the interference generated by their desorption and ionization, OCT matrices are nevertheless quite regularly encountered in SALDI‐MSI (~ 10% of the samples reported in Table 3). Compared with OCT matrices, gelatin provides a much cleaner signal background (Chughtai & Heeren, 2010; Phan et al., 2016).

### 3.2 Cryosectioning

Once stabilized, samples are generally cut in thin and flat microsections (required for MSI analyses) using a cryo‐microtome. In SALDI‐MSI analyses, section thickness is usually chosen between 10 and 20 µm. Indeed, thinner sections tear easily and thicker sections, although easier to manipulate, require longer drying‐time, which can cause cracking and warping of the sections. Yet, some authors reported sections down to 3–5 µm, enabling to better visualize histological features and required in the “deposition” method as it will be further explained in Section 3.5. Sections up to 30‐µm thick were also reported, suitable in the “imprinting” method (See Section 3.5). To perform MSI analyses, the sample sections must be mounted on an electrically conductive target plate, to properly extract the ions produced at the sample surface. Hence, thicker sections might not be conductive enough and thin sections are often preferred. The conductive support is usually either an Indium Tin Oxide (ITO)‐coated glass slide or a nanostructured substrate for SALDI‐MSI.

### 3.3 Selection of the nanosubstrates for SALDI‐MSI

Once the sample has been selected, collected, and prepared, the next step is to consider the selection of the appropriate SALDI nanosubstrate based on the target analytes and applications. Back in 1988, Tanaka et al. already defined characteristics that the materials have to present to be suitable to assist the LDI process (Tanaka et al., 1988). Among these features are a strong absorption in the UV range, allowing efficient absorption of the laser energy, and a low heat capacity and a large surface area per volume unit, both ensuring rapid heating, highly localized and uniform energy deposition (Morosi et al., 2013; Northen et al., 2007). In this context, nanomaterials, displaying the ideal characteristics, have been attracting considerable attention and their development led to the emergence of SALDI‐MS. Moreover, SALDI nanosubstrates have additional interesting physicochemical properties compared with organic matrices, such as low chemical background in LDI‐MS, non‐volatility (they are thus stable in vacuum conditions for MS imaging experiments) (Hansen et al., 2019) and their large surface area offers high molecular loading capacities (>1000 small molecules per nanoparticle) (Abdelhamid, 2018).

Since the first implementations of SALDI‐MS, a great variety of nanomaterials and nanostructured surfaces have been tested and reported as SALDI nanosubstrates, with varying degrees of success (Law & Larkin, 2011). However, in this section, we only focus on the different nanosubstrates used in SALDI‐MS imaging applications, as the nature of the nanosubstrates used in “general” SALDI‐MS experiments has already been depicted in many other SALDI‐MS reviews (Abdelhamid 2019, 2018; Chiang et al., 2011; Lu et al., 2017).

#### 3.3.1 Chemical composition of the nanosubstrates

**Figure 9:**
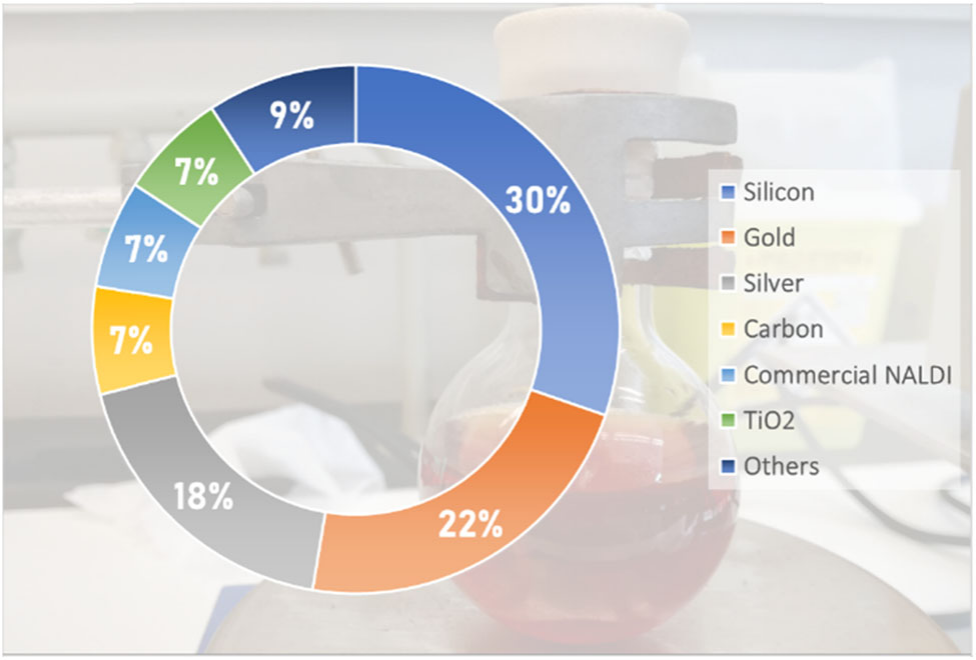
Compositions of the nanosubstrates used in SALDI‐MSI [Color figure can be viewed at wileyonlinelibrary.com]

Nanosubstrates with various compositions have been used in SALDI‐MSI, as shown in Figure 9. Nanosubstrates are mainly based on silicon, gold, and silver but TiO_2_ and carbon‐based nanosubstrates as well as the commercial NALDI™ plate (Bruker Daltonics) are also quite usually met in SALDI‐MSI.

**Figure 10:**
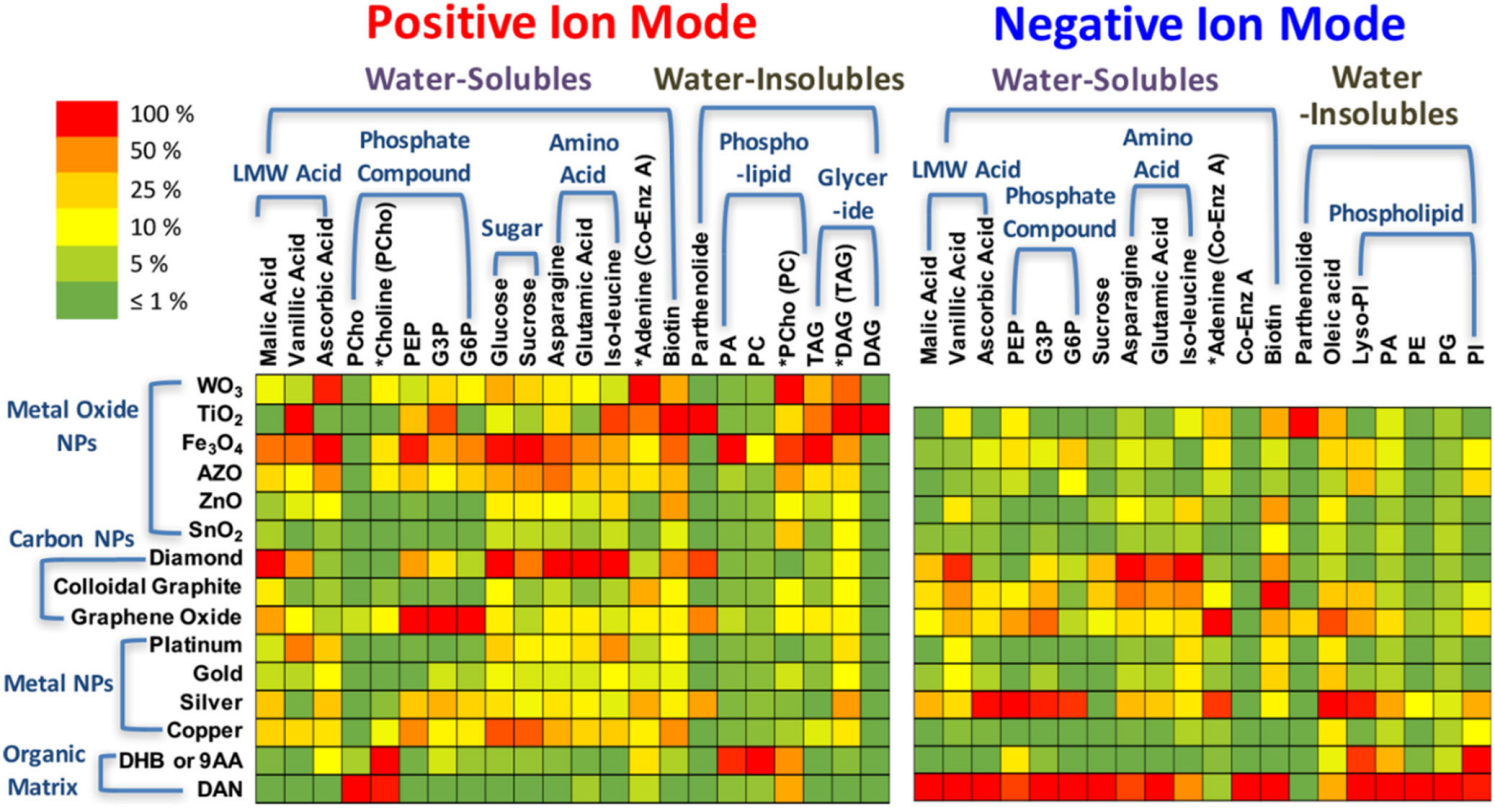
Summary of nanoparticle screening for small molecule metabolite analysis. Ion signals are normalized to the highest ion signal for each analyte and shown as a heat map. DHB and DAN were used for positive ionization mode and 9‐AA and DAN were used for negative ionization mode. Reprinted with permission from Yagnik (2016). © 2016 American Chemical Society [Color figure can be viewed at wileyonlinelibrary.com]

This variety of compositions can be partly explained by the preferential desorption/ionization of some specific classes of analytes with certain elements, as shown in Figure 10. Some nanomaterials are known for their high natural affinity for specific functional groups and can thus be efficiently used to specifically analyze certain classes of analytes (Yagnik et al., 2016). There are several well‐known examples; one of the best known is the affinity of gold and silver for sulfur‐containing compounds (including thiols) (Arakawa & Kawasaki, 2010). Another widely exploited example is the ability of silver (a strong Lewis acid) to form weak charge transfer complexes with double bonds (Muller et al., 2017), which makes it selective for long‐chain unsaturated hydrocarbons (Arakawa & Kawasaki, 2010; Dufresne et al., 2013), which are usually difficult to ionize with conventional MALDI‐MS (Schnapp et al., 2016), and for aromatic compounds by forming Ag adducts (Ozawa et al., 2016). Several authors took profit of this unique affinity to image the distributions of cholesterol and other lipids as well as olefins in rodent brain sections, an organ rich in lipids with important biological functions (Dufresne et al., 2013; Guan et al., 2018; Muller et al., 2017; Yang et al., 2020). Lipids species forming silver adducts were also imaged in other rodent organs such as rat kidney (Dufresne et al., 2013; Muller et al., 2015), rat heart (Jackson et al., 2014) and mouse retinal sections (Hayasaka et al., 2010). Alternatively, silver affinity toward lipids was used to image butterfly hindwing and fingermarks (Schnapp et al., 2016). Gold nanoparticles, besides their affinity for sulfur‐containing compounds, also offer selective ionization for some lipids especially triacylglycerides and small peptides, such as des‐acyl ghrelin in fly brain (Phan et al., 2016) and glycosphingolipids in mouse brain (Goto‐Inoue et al., 2010). Metal oxide nanoparticles are also known for specific affinities. For example, ZnO nanoparticles have an affinity for amines, whereas TiO_2_ nanoparticles can selectively ionize enediol compounds (Arakawa & Kawasaki, 2010). TiO_2_ nanoparticles are also known for their affinity for phosphorylated compounds (Pilolli et al., 2012).

Figure 10 also clearly shows the affinities of different nanosubstrates toward some of the low molecular weight metabolites, experimentally demonstrated (Yagnik et al., 2016). For example, TiO_2_ nanoparticles are more inclined to detect water insoluble metabolites, especially parthenolide (a terpene) in positive ionization mode. Carbon‐based nanoparticles are also characterized by specific affinities: diamond nanoparticles work well with sugars and amino acids, in both ionization modes, while graphene oxide nanoparticles are particularly efficient with phosphate compounds in positive ionization mode. Complementarily, silver nanoparticles allow the sensitive detection of phosphate compounds in negative ionization mode. Pt usually interacts with alkyl carboxylic acid and alkylamine (Kawasaki et al., 2009), which may justify its affinity for amino acids. The carboxylic functional group can also link with Fe (Kawasaki et al., 2009). The intrinsic affinities of these different nanosubstrates for various types of specific analytes also shows the potential of a combination of several nanosubstrates in one single experiment. A thermal desorption model has been developed to explain the different SALDI‐MS efficiencies (Yagnik et al., 2016). However, the influence of other parameters including hydrophobicity‐hydrophilicity and electrostatic properties of the surface still have to be investigated to understand the preferential desorption/ionization observed for the nanosubstrates. Indeed, without a better understanding of the SALDI fundamental mechanisms (see Section 2.3), the selection of the nanosubstrate will remain purely empirical.

As also shown in Figure 10, the composition of the nanosubstrate will usually dictate the choice of the ionization mode (either positive or negative). For example, the analysis of vanillic acid will be preferentially performed in negative ionization mode with diamond nanoparticles and in positive ionization mode with TiO_2_ nanoparticles. It is however worth to note that the most common nanomaterials, such as silver‐ and gold‐based nanoparticles (Müller et al., 2020), can work in both ionization modes, either positive or negative for the analysis of various small molecules (Abdelhamid, 2018). In another study, gold and silver‐based nanoparticles are clearly efficient in both ionization modes for soluble and insoluble water compounds (Hansen et al., 2019). Porous TiO_2_ film immobilized with gold nanoparticles also exhibit high performance in dual polarity analyses (Wang et al., 2020). On the contrary, usual MALDI matrices such as DHB or 9‐AA are respectively used in positive and negative ionization modes. Figure 10 also indicates that 1,5‐diaminonaphthalene (DAN) is more efficient than the nanoparticles in the negative ionization mode.

The SALDI mass spectra of the samples can also be internally mass calibrated using the nanosubstrate ions or cluster ions (e.g., Au_n_^+/−^ and Ag_n_^+/−^ clusters) (Kolárova et al., 2017; Prysiazhnyi et al., 2019; Ràfols et al., 2018). Indeed, in most cases, the nanosubstrates used in SALDI‐MSI are also ionized and associated with intense signals. Nevertheless, the ionization of the LDI‐assisting substrate in SALDI‐MSI does not significantly increase the chemical background of the spectra in the low *m/z* values, which is not the case in MALDI‐MS. Moreover, recently developed chemometric approaches enable to annotate the signals attributed to the nanosubstrate in SALDI‐MSI and thus, to clean up the mass spectra from the LDI‐assisting substrate signals (Baquer et al., 2020). In this context, silver‐based nanosubstrates are particularly adapted thanks to the two abundant and stable natural isotopes of silver, ^107^Ag (51.839%) and ^109^Ag (48.161%), which can be distinguished in the mass spectrum. Several silver clusters can thus be identified based on exact mass measurements and their characteristic isotope patterns. Additionally, silver is not naturally abundant in biological samples, such as tissues or cell cultures (Muller et al., 2015). Therefore, one can be ensured that the image of the analytes as silver adducts (thanks to the ionization of silver from the nanosubstrate itself) represents the natural distribution of the analytes. This is not necessarily the case for Na^+^ or K^+^ adducts. Indeed, these ubiquitous alkali ions are naturally abundant and thus, the image of the alkali cationised adducts may be more dependant of the distribution of Na^+^/K^+^ ions across the imaged samples (Muller et al., 2015). Moreover, abnormal Na^+^ and K^+^ distributions may result from an alteration of the ATP metabolism underlying a disease (Guan et al., 2018). However, close attention has to be paid to the SALDI‐MSI data processing due to the high heterogeneity in the formation of adducts between biological compounds from the sample and silver cations (Baquer et al., 2020).

#### 3.3.2 Morphology of the nanosubstrates

The majority of the nanosubstrates employed in SALDI‐MSI are either nanostructured solid surfaces (~40% of the samples reported in Table 3) or colloidal nanomaterials (~40% of the samples reported in Table 3) sprayed at the sample surfaces (see Section 3.5). Sputtered metal nanoclusters are also commonly encountered in SALDI‐MSI (~20% of the samples reported in Table 3). Besides this general appearance, the fine nanostructure of the nanosubstrate can also be described. The morphology of the nanosubstrate (e.g., size, porosity, type of nanostructure) is of prime importance as it significantly affects the desorption/ionization efficiency in SALDI‐MS (Zhu et al., 2020). For instance, it has been demonstrated that the ionization/desorption processes are greatly impacted by the roughness/porosity of the nanostructure (Law, 2010) and, in particular, by the pore depth (Xiao et al., 2009). The size of TiO_2_ particles in monoliths was also found to affect the detection of intact lipids and their fragmentation (Wu et al., 2017). The appropriate nanosubstrate morphology can also improve the sample preparation. For example, a novel porous aluminum oxide slide has been recently developed to drastically minimize lipid delocalization and ion suppression effects (Fournelle et al., 2020).

In the context of SALDI‐MSI, colloidal nanomaterials are almost exclusively used in the form of colloidal graphite (or graphene oxide) or colloidal nanoparticles of different diameters, usually comprised between 2 and 10 nm. Nanoparticles with diameters higher than 30 nm have also been reported in SALDI‐MSI (Müller et al., 2020; Tseng et al., 2017; Wang et al., 2020).

**Figure 11:**
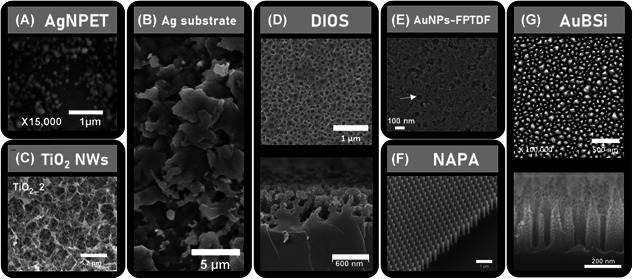
SEM images of (A) AgNPET. Reprinted with permission from Niziol et al. (2013). © 2012 Elsevier B.V. (B) etched Ag substrates. Reprinted with permission from Schnapp et al. (2016). © 2016 Elsevier Inc. (C) TiO_2_nanowires (NWs). Reprinted from Dutkiewicz et al. (2019) (CC BY‐NC‐ND 4.0). (D) DIOS nanosubstrate, top view (top) and cross‐section (bottom). Reprinted with permission from Guinan et al. (2015b). © 2015 The Royal Society of Chemistry. (E) Functionalized porous TiO_2_film immobilized with gold nanoparticles (AuNPs‐FPTDF). Reprinted with permission from Wang et al. (2020a). © American Chemical Society. (F) NAPA platform. Reprinted with permission from Morris et al. (2015). © 2015 The Royal Society of Chemistry. (G) Gold‐coated black silicon substrate (AuBSi). Reprinted with permission from Iakab et al. (2020). © 2020 American Chemical Society

The fine structure of the solid nanosubstrates is more diversified, as shown in Figure 11, ranging from highly engineered, controlled and uniform nanostructures to more randomly organized structures. For example, the solid nanosubstrates can be prepared by coating a target plate with metallic nanoparticles. The research team of T. Ruman has developed AgNPET (Figure. 11A) (Niziol et al., 2013, 2019, 2020) and AuNPET (Misiorek et al., 2017; Niziol et al., 2016; Sekula et al., 2015a), by coating a steel target with ~100 nm of AgNPs and AuNPs, respectively. Metal targets can also be directly etched to form Ag etched substrates, for example (Figure 11B) (Schnapp et al., 2016). A titanium target has also already been etched through an inexpensive modified hydrothermal process to form TiO_2_ nanowires (Figure 11C) (Dutkiewicz et al., 2019). Nanowires also constitute the silicon nanostructure of the NALDI™ plate used in various SALDI‐MSI studies (Krasny et al., 2015; Skriba & Havlicek, 2018; Tata et al., 2012, 2014; Vidova et al., 2010). In DIOS, the nanostructured surface is produced via the etching of a silicon wafer, leading, for instance, to ordered nanocavity arrays (Liu et al., 2007). DIOS nanosubstrates are employed in several works (Guinan et al., 2015a; Ronci et al., 2012; Rudd et al., 2015). Figure 11D shows a DIOS nanosubstrate characterized by a pore diameter of ~80 nm and depth of ~450 nm (Guinan et al., 2015b). The etched silicon surface was further oxidized and finally silanised. The NIMS chips share a common fabrication procedure with DIOS platforms, except that the silicon etched surface of the NIMS chips are further coated with an fluorinated initiator solution (e.g., BisF17, Bis(heptadecafluoro‐1,1,2,2‐tetrahydrodecyl)tetramethyldisiloxane) (Woo et al. 2008). NIMS chips are mainly used by G. Siuzdak and coworkers (Lee et al., 2012; O'Brien et al. 2013; Patti et al., 2010a, 2010b; Yanes et al., 2009). In SPILDI (or SP‐LDI), the solid substrate is simply a silica TLC plate (De Oliveira et al., 2014; Ferreira et al., 2014). Besides silicon‐based nanosubstrates, TiO_2_ has also been used as a porous nanostructure covered with AuNPs (Figure 11E) (Wang et al., 2020). Finally, some nanostructured solid substrates used in SALDI‐MSI are more engineered leading to more complex nanostructures. For example, in NAPA‐LDI, the nanosubstrate is composed of nanopost arrays, also prepared from silicon wafers. Figure 11F shows the highly ordered and uniform NAPA platform with silicon nanoposts characterized by an average height of 1100 nm, an average diameter of 150 nm and an average periodicity of 337 nm) (Morris et al., 2015). NAPA platforms have been developed and are still commonly used by the research group of A. Vertes (Fincher et al. 2019a, 2019b, 2020a, 2020b; Stopka et al., 2016). The gold‐coated black silicon substrates (AuBSi) (Figure 11G) developed by Iakab et al. are also constituted of an array of vertical silicon nanopillars or spikes (height ~ 300 nm, diameter ~60 nm and spacing ~45 nm) (Iakab et al., 2020). These nanopillars constitute the black silicon (BSi) nanostructure, which was further coated by a 10 nm layer of gold nanoislands or nanoparticles (Iakab et al., 2020).

### 3.4 Functionalization of the nanosubstrates

When intricate samples (e.g., biological tissue section, bacterial culture, etc.) are interrogated by LDI‐MSI techniques, including both SALDI and MALDI, the ionization of a great number of “unwanted” molecules occurs along with the ionization of the target analytes. This is a major problem in MSI as it may significantly complexify the mass spectra and lead to the suppression of the ions of interest. In this context, SALDI‐MSI offers a particularly valuable advantage over MALDI‐MSI. Indeed, the desorption and ionization processes in SALDI‐MSI are known to be affected by the nanosubstrate morphology and chemical nature (which both define the physicochemical properties of the nanosubstrate). Hence, by tailoring these nanosubstrate parameters, by grafting specific ligands on the nanosubstrate surface for example, the specificity and sensitivity of the analysis can be greatly enhanced. The endless possibilities of surface functionalization have enormous potential as they allow to get around some significant disadvantages of the LDI techniques (Arakawa & Kawasaki, 2010), especially regarding the imaging of non‐abundant species. For instance, trace enrichment can be performed thanks to the interaction of the analyte with the ligand attached to the nanosubstrate surface. SALDI‐MSI also offers another approach in which nanomaterials can act as “mass reporters.” In this targeted approach, the signal that is monitored is produced either by the ions or clusters of the nanomaterials or by a ligand grafted to the nanomaterials and not by the analytes. In that case, signal detection can be greatly enhanced in complex samples. Thus, SALDI‐MSI is particularly valuable for the analysis of low‐abundant compounds in an imaging context where the sample treatment and enrichment of the analyte of interest are limited.

#### 3.4.1 Functionalized affinity probes

**Figure 12:**
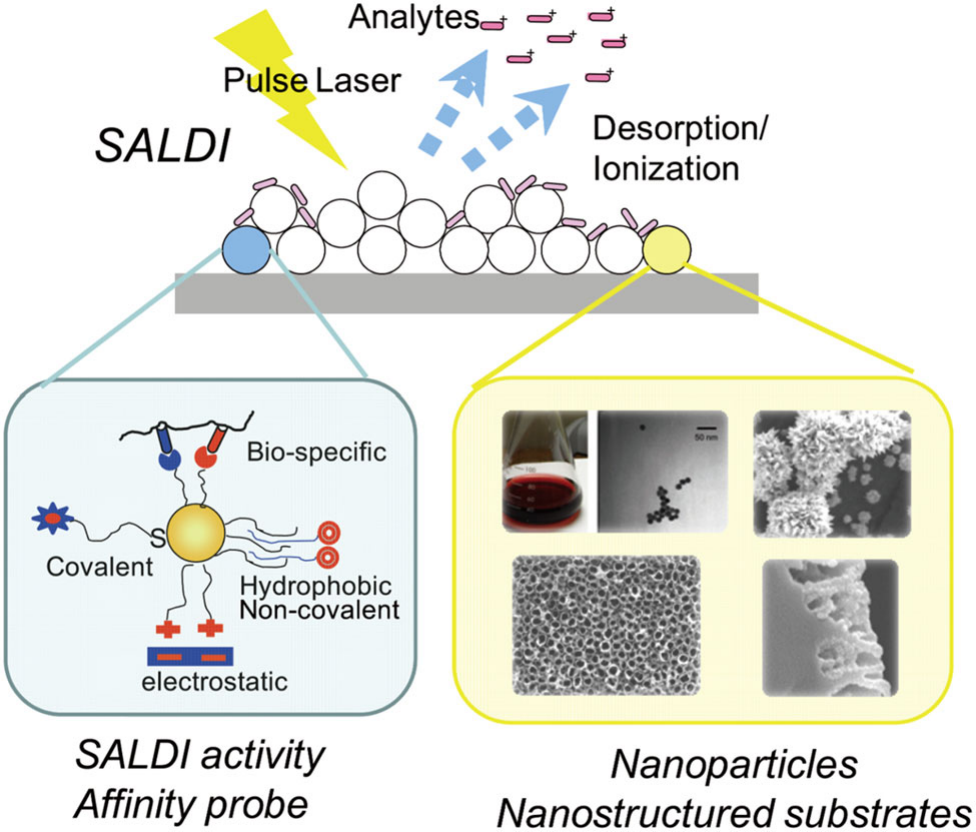
Schematic representations of SALDI‐MS using various nanoparticles and nanostructured substrates and an affinity probe. Reprinted with permission from Arakawa et al. (2010). © 2010 The Japan Society for Analytical Chemistry [Color figure can be viewed at wileyonlinelibrary.com]

While the specificity of an imaging experiment can be enhanced by the natural affinity of the surfaces for various adsorbed compounds, it can also be increased by the functionalization of the nanosubstrate surface with diverse targeting/capturing ligands having a high affinity for particular analytes through specific interactions (e.g., hydrophobic, electrostatic, bio‐specific, and so forth (Arakawa & Kawasaki, 2010), as shown in Figure 12.

For example, thiol‐terminated ligands can be immobilized on various surfaces including Au, Ag, Pt, and Fe via the formation of metal‐S bonds (Kawasaki et al., 2009). Porous silicon can also be functionalized, notably by avidin‐biotin chemistry or through antibody immobilization (Ocsoy et al., 2013). Antibodies have high specificity toward their antigens but are expensive and only disposable in a small amount on the nanosubstrate surface due to their bulky size (Lai et al., 2015). Aptamers were therefore proposed as an alternative as they have a smaller molecular size and can be easily synthesized (Lai et al., 2015). Moreover, aptamers offer a wide range of targets, including DNA or RNA sequences, large proteins, and even biological cells (Ocsoy et al., 2013). In an imaging context, Dutkiewicz et al. improved the selectivity toward *Catharanthus roseus* secondary metabolites called vinca alkaloids by functionalizing TiO_2_ nanowires with perfluorooctyl chain (Dutkiewicz et al., 2019). The specificity toward specific analytes can also be enhanced by modifying the physico‐chemical properties of the nanosubstrate surface through functionalization (Iakab et al., 2020; Wu et al., 2017). For instance, AuBSi substrates (i.e., black silicon substrates coated with AuNPs) were functionalized with hydrophilic and hydrophobic groups, stimulating specific interactions between the nanosubstrate surface and the analytes (Iakab et al., 2020). The surface of TiO_2_ monoliths was also already modified with dopamine ligands to enhance imaging selectivity and sensitivity toward Lewis basic compounds, such as fatty acids, cholesterols, ceramides, diacylglycerols, and phosphatidylethanolamine (Wu et al., 2017). The dopamine ligands notably led to higher surface pH, which improved the detection of phospholipids (Wu et al., 2017).

However, upon laser irradiation, the surface ligands may also desorb and ionize and the formation of metal cluster ions may occur as well. These two phenomena can lead to the suppression of the analyte ions.

#### 3.4.2 Mass‐tag reporters

**Figure 13:**
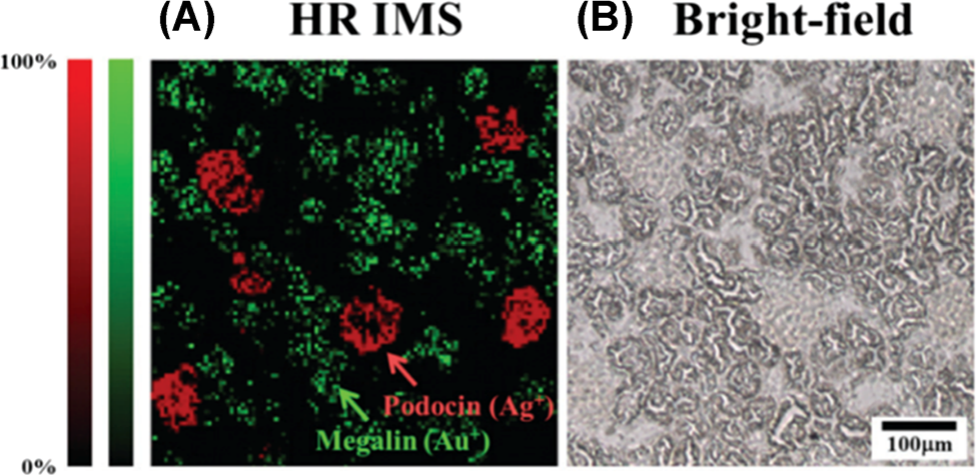
(A) Duplex MSI result showing the distribution of megalin (reported by Au^+^) and podocin (reported by Ag^+^) in the cortex region, and (B) the corresponding bright‐field optical micrograph. Reprinted with permission from Cheng et al. (2019b). © 2019 The Royal Society of Chemistry [Color figure can be viewed at wileyonlinelibrary.com]

To overcome the ion suppression of the targeted analytes or to analyze low abundant species, monitoring the signals produced by the desorption/ionization of the surface ligands and/or nanosubstrate cluster ions is usually more convenient. This indirect targeted approach provides higher sensitivity relative to the direct approach consisting in analyzing the signal belonging to the analytes (Unnikrishnan et al., 2016). In the “mass‐tag” approach, the nanosubstrates are functionalized to bind specifically with a molecule of interest (e.g., proteins, lipids, membrane receptor,…). Then, the signals of the ions from the nanosubstrate (e.g., Ag or Au cluster ions) and/or of the ligands, which are sometimes called “mass‐tag reporters,” “mass barcodes,” “signal tags,” or “signal reporters” are monitored. The detection of the signal reporter in the mass spectrum indicates the presence of the targeted molecule in the sample and the spatial distribution of the molecule of interest can be determined by mapping the mass‐tag reporter ion intensity (Cheng et al., 2019b). In addition, the use of the “mass‐tag approach” enables signal amplification, particularly interesting for the analysis of minor analytes. For example, a single 12.9 nm gold nanoparticle is composed of about 64,000 gold atoms (Liu et al., 2013) allowing the amplification of the MS signal for several orders of magnitude compared with the signal proper to the ionization of the targeted analyte. The mass‐tag reporters have also notably been used to detect molecules characterized by low ionization efficiency and easy fragmentation, such as DNA (via DNA hybridization with a complementary sequence grafted on the nanosubstrate), which is still challenging with conventional MS (Pilolli et al., 2012). This targeted approach has also already been applied for imaging experiments. For example, Cheng et al. used antibody‐conjugated gold and silver nanoparticles to control the filtration and reabsorption of two proteins, megalin and podocin, in the excretion system of mouse kidney (Cheng et al., 2019b). As shown in Figure 13, the use of nanoparticles composed of distinct elements allows multiplex SALDI‐MSI. Cheng et al. mapped the signals of Au^+^ and Ag^+^ to localize megalin and podocin, respectively (Figure 13).

**Figure 14:**
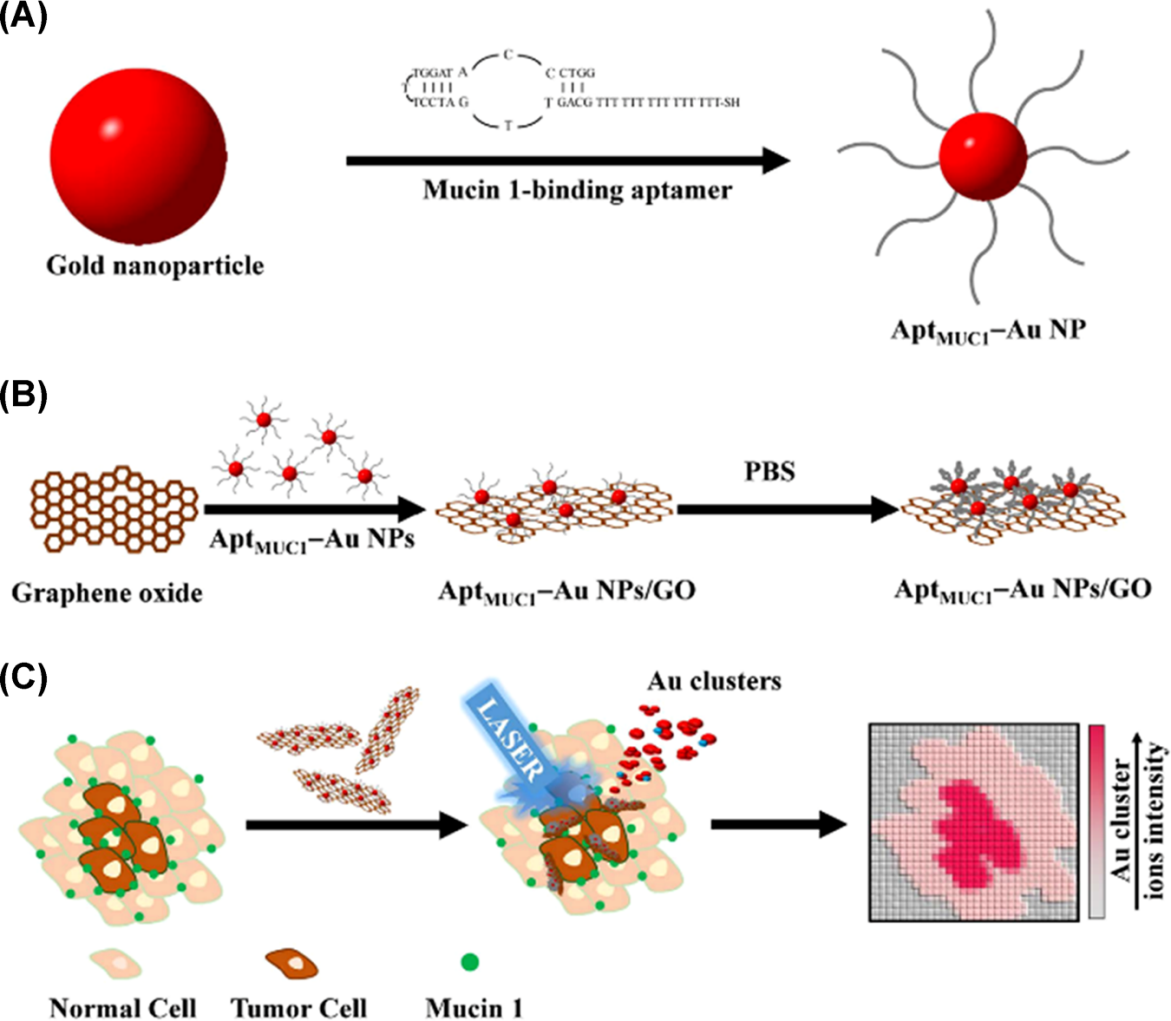
Schematic representation of (A) the preparation of MUC1‐binding aptamer–modified gold nanoparticles (AptMUC1–AuNPs) and (B) their conjugation to graphene oxide (AptMUC1–AuNPs/GO) for (C) tumor tissue imaging by monitoring Au cluster ions. Reprinted from Huang et al. (2015) (CC‐BY 4.0) [Color figure can be viewed at wileyonlinelibrary.com]

Still in the biomedical imaging field, Huang et al. based their approach on Mucin1 (MUC1)‐binding aptamer (AptMUC1) as a targeting agent (Huang et al., 2015). MUC1 is a large transmembrane glycoprotein representing an attractive cancer biomarker overexpressed in most adenocarcinomas (Huang et al., 2015). They conjugated AptMUC1 with gold nanoparticles and immobilized these nanoprobes (AptMUC1‐AuNPs) on graphene oxide (AptMUC1–AuNPs/GO). The engineered AptMUC1‐AuNPs/GO were found to effectively bind to MUC1 units on tumor cell membranes and can be localized by monitoring Au cluster ions ([Au_n_]^+^; n = 1–3). The procedure is summarized in Figure 14.

**Figure 15:**
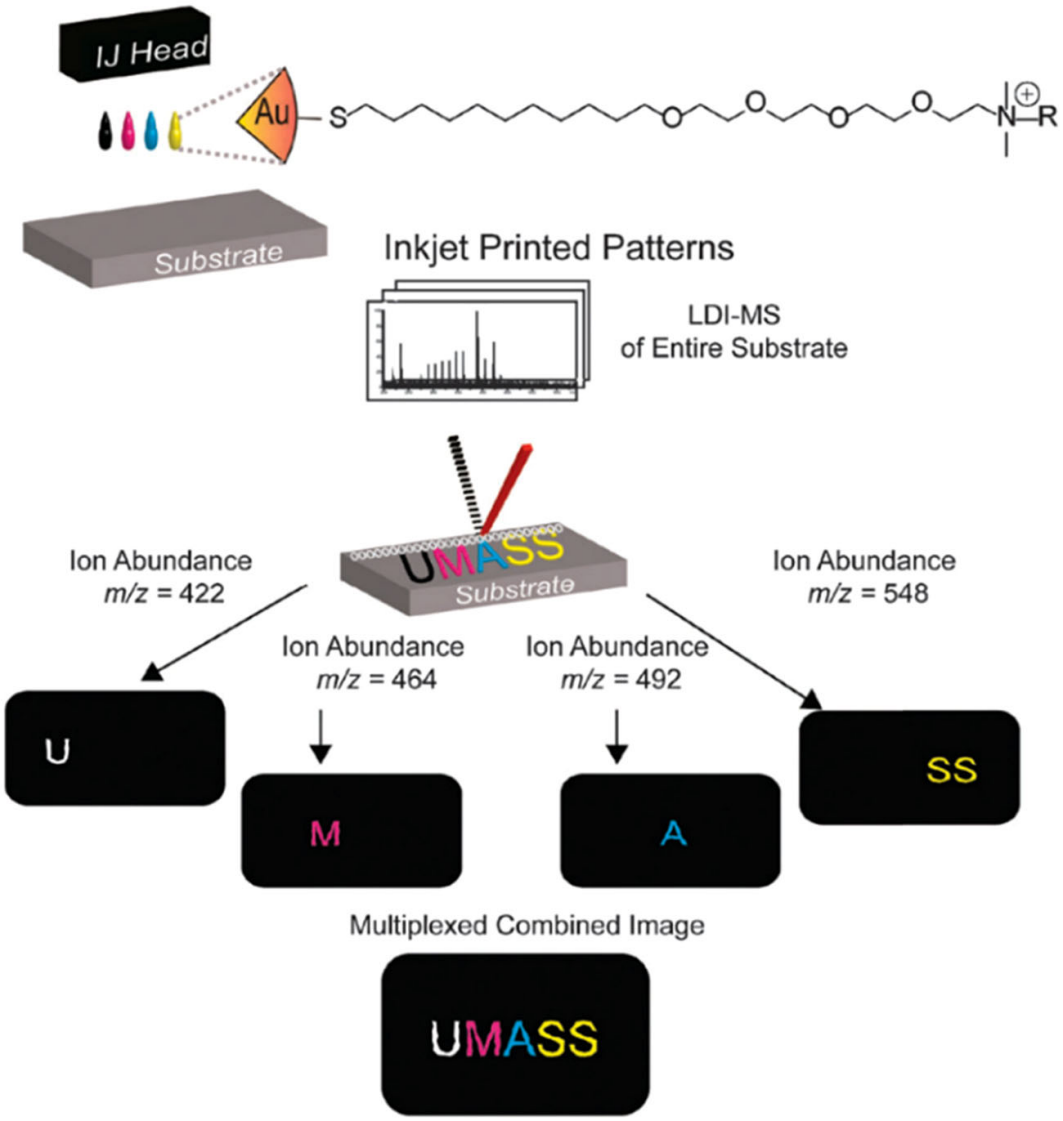
Anticounterfeiting mass barcoding strategy. Reprinted from Creran et al. (2012). © 2012 The Royal Society of Chemistry (CC‐BY 3.0) [Color figure can be viewed at wileyonlinelibrary.com]

In a completely different context (i.e., untargeted imaging), Creran et al. functionalized gold nanoparticles with surface ligands characterized by unique structures and therefore unique MS fingerprints for anticounterfeiting applications (Creran et al., 2012). In their work, the functionalized nanoparticles were not used to target a specific analyte but were patterned onto a surface by inkjet printing, providing discernible design through SALDI‐MSI, as shown in Figure 15.

More information on affinity probes and functionalized nanoparticles in SALDI‐MS analyses can be found in several other reviews. For example, Unnikrishnan et al. reviewed the applications of functionalized gold nanoparticles for signal amplification in biosensing and bioimaging (Unnikrishnan et al., 2016), Zhu et al. reviewed the functionalization of nanoparticles for selective extraction and concentration, improved ionization and mass “barcodes” applications (Zhu et al. 2009) and Arakawa et al. reviewed the use of nanoparticles as affinity probes (Arakawa & Kawasaki, 2010).

### 3.5 Deposition/utilization of the nanosubstrates

SALDI‐MSI represents nowadays an interesting and promising alternative to MALDI‐MSI as the use of nanostructured substrates enables to get around most of the matrix‐related issues encountered in MALDI (Arakawa & Kawasaki, 2010; Pilolli et al., 2012). Moreover, SALDI‐MSI offers easier sample preparation compared with MALDI‐MSI, as it does not require the co‐crystallization of the organic matrix with the analytes (Picca et al., 2017; Sekula et al., 2015b). Indeed, for SALDI‐MSI, the nanosubstrates are, in most cases, deposited as a homogeneous and regular coating by spraying or sputtering. Moreover, no spraying is required in the imprinting and deposition methods as the solid nanosubstrate is used as it is. Thus, SALDI‐MSI experiments are generally characterized by a higher reproducibility (Krasny et al., 2015) and higher spatial fidelity and resolution than conventional MALDI‐MSI (Lopez de Laorden et al., 2015). In turn, this leads to more accurate quantitative analyses in SALDI‐MS (Qiao & Liu, 2010), which opens new opportunities in terms of quantitative imaging analyses (Cazier et al., 2020).

**Figure 16:**
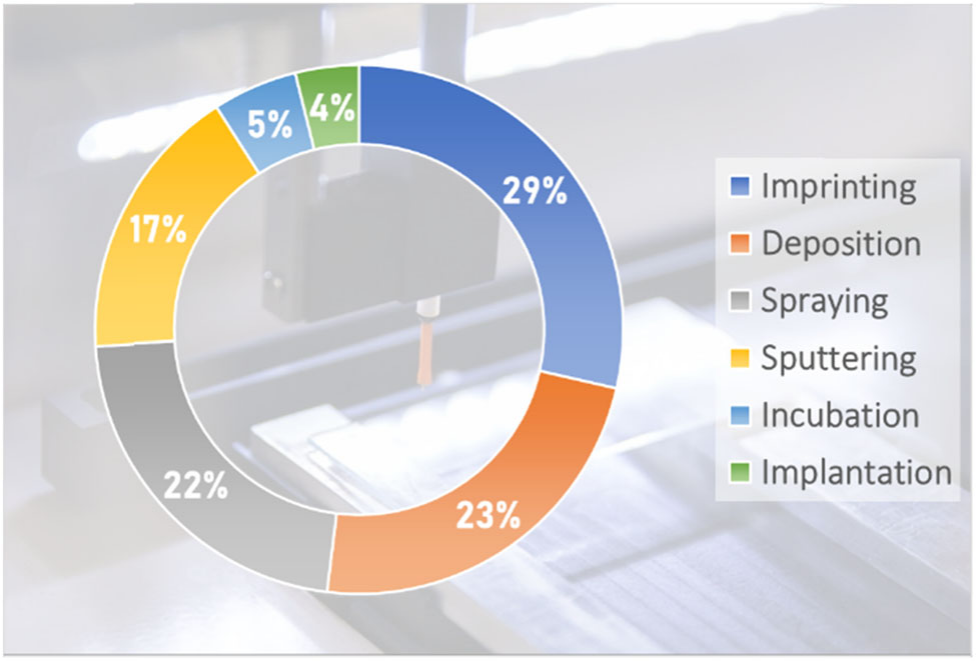
Deposition/application method of the nanosubstrates used in SALDI‐MSI [Color figure can be viewed at wileyonlinelibrary.com]

Currently, there are four major ways to use the nanosubstrates in SALDI‐MS imaging, which are the “imprinting method,” the “spraying method,” the metal sputtering, and the sample deposition on the nanosubstrate as shown in Figure 16. The implantation of metallic nanoparticles in the sample can also be used to a lesser extent. In the “mass‐tag” approach, samples are also sometimes simply incubated with the nanoprobes. This method will not be discussed in this section.

#### 3.5.1 Spraying method

Spraying methods were among the first techniques to be introduced for SALDI‐MS imaging and allow a fairly good control of the nanosubstrates deposition. In this method, the colloidal nanosubstrates are initially in suspension in an appropriate solvent (such as methanol or acetonitrile, which quickly evaporate) preserving the stability of the colloid. Then, homogeneous layers of the nanosubstrates are sprayed on the sample, either manually or automatically. Several devices can be used to coat the sample such as manual pneumatic spray systems (artist paintbrush, thin layer chromatography venturi flask, airbrush, etc.) or automatic spray systems (McLaughlin et al., 2020), which allow a better control and reproducibility of the spraying conditions (debit, temperature, and deposition pattern) (Chaurand, 2012). However, the use of solvent in the spraying method could have limitations in MSI as the involvement of a solvent for the coating of the nanomaterials could possibly cause the delocalization of the small molecules in the samples (Tang et al., 2011b). Moreover, the deposition of a uniform nanosubstrate layer may be challenging due to the aggregation of the nanomaterials. Also, during the deposition of several nanosubstrate layers, the diffusion of the nanosubstrates can lead to their inhomogeneous distribution through the sample surface (Kawasaki et al., 2012). Therefore, to favor a fast evaporation of the solvent, the coating is deposited as a succession of thin films (typically ten to a few dozens).

#### 3.5.2 Imprinting method

The imprinting method was initiated by the work of Vidova et al. (Vidova et al., 2010) and is now largely carried out by Ruman's group through the development and use of their metal (i.e., Au and Ag) nanoparticle enhanced targets (AuNPET and AgNPET) (Niziol et al., 2019, 2016; Sekula et al., 2015b, 2015c). In the imprinting method, the sample is placed in direct contact with the nanostructured surface to enable direct surface transfer of the chemical compounds from the sample to the nanostructure. In this method, the sample is removed before the analysis, leaving a molecular imprint of the surface. A variant of the imprinting method, called “Replica‐Extraction‐Transfer,” can also be employed and consists in (1) the use of a solvent‐laden semisolid (e.g., gel) to extract metabolites from a microbial sample, such as a biofilm or agar culture and (2) the “replication” of the metabolites by imprinting the gel onto the nanosubstrate (Louie et al., 2013).

While the imprinting method is extremely simple and does not require particular instrumentation, it is vulnerable to smudging of the spatial details during the imprinting step, which limits the spatial resolution (Rafols et al., 2018) and can lead to misinterpretation of the MSI data (Fournelle et al., 2020). Moreover, the imaging of low abundant metabolites is not always feasible. Some issues may also arise from the inefficiency in transferring the analytes on the nanosubstrate by imprinting. In particular, the properties of the substrate surface (e.g., hydrophobicity) significantly determine which analytes will be imprinted on the surface (Iakab et al., 2020). Moreover, the surface morphology also affects the imprinting performance, notably in terms of sensitivity. For example, in NAPA, the sensitivity of small molecules (<2000 Da) has been found to be higher with increased surface porosity whereas lower porosity favors the analysis of largest molecules (Muthu et al., 2017).

#### 3.5.3 Deposition

Another method generally used with solid nanosubstrates is the simple deposition of the sample on the nanostructured surface. In the deposition method, the sample is deposited and kept over the nanosubstrate during the analysis. This method is generally employed in the SALDI variant referred as NIMS (i.e., nanostructure‐initiator MS). Cells (e.g., cell cultures [Stopka & Vertes 2020], bacteria [Dutkiewicz et al., 2019], embryos [Ferreira et al., 2014], and cancerous cells [O'Brien et al., 2013]) are also imaged following their deposition on the substrate. The disadvantage of this method in the case of tissue imaging is that it requires very thin tissue sections (<5µm), which are difficult to prepare and usually require the embedding of the sample in a protective material. Thicker sections are generally characterized by lower ionization efficiency and may affect the conductivity. Moreover, imaging artifacts may be observed due to nonuniform desorption/ionization of the analytes within the sample (Ronci et al., 2012). Indeed, the ionization of the analytes occurs when the laser beam energy is absorbed by the underlying nanosubstrate and then transferred to the analytes (Ronci et al., 2012). However, histologically different regions of a tissue section, for example, may behave differently upon laser irradiation.

#### 3.5.4 Sputtering method

One of the latest methods developed for SALDI‐MSI is the sputtering method, greatly carried out by the group of Chaurand (Dufresne et al., 2013, 2016; Lauzon et al., 2015). The sputtering method allows the deposition of thin homogeneous layers of pure metal nanoclusters (Dufresne et al., 2013). During sputtering, particles of a solid elemental and high purity metallic material (generally called the “target” material or source) are extracted from the metal surface, which is bombarded by a beam of charged gas particles or with a plasma, in vacuum. Sputtering deposition usually employs an argon plasma as argon is a noble gas that does not chemically react with the target source.

The thicknesses of the metal layers used by the different authors are quite variable and depend on the sample type, on the analytes and on the metal used. For example, Dufresne et al. used 28 ± 3 nm layer of Au to image the distribution of triacylglycerols in mouse liver and rabbit adrenal gland tissues (Dufresne et al., 2016), 23 ± 2 nm of Ag to analyze lipids in mouse brain (Dufresne et al., 2013) and 16 ± 2 nm of Ag to analyze lipids in mouse kidney, liver and testis (Dufresne et al., 2013). Lauzon et al. deposited 14 ± 2 nm Ag layer to image the molecular composition of fingerprints (Lauzon et al., 2015). Ozawa et al. sputtered 10 nm Pt films to image insecticides in plant leaves (Ozawa et al., 2016). Yang et al. used 28 nm thick Ag layer to image cholesterol and olefins in mouse brain (Yang et al., 2020). A thinner Au layer (4 nm) was also employed by Tang et al. for the histological analysis of animal tissues (Tang et al., 2011b).

This solvent‐free approach allows the rapid and uniform nanosubstrate coating. This approach also allows to avoid the analyte delocalization (Rafols et al., 2018), thus improving the lateral resolution. Moreover, the sputtering method eliminates the aggregation problem of colloidal suspensions (Hansen et al., 2019). The sputtering of a metal layer also renders the surface conductive, which allows the imaging of samples on nonconductive surfaces (Lauzon et al., 2015) as well as the imaging of thick and nonconductive samples (Ozawa et al., 2016), which is a benefit over MALDI‐MSI.

However, despite its advantages, the sputtering method remains less commonly employed than the other previously discussed methods, as shown in Figure 16. This may be due to the need for specialized sputtering systems as well as technical skills (Yang et al., 2020). Indeed, the sputtering method generally requires the precise control of the optimal sample‐dependant coating thickness (Schnapp et al., 2016). As an example, too long coating times (and therefore too thick metal layer) may prevent the proper desorption/ionization of the underlying analytes (Dufresne et al., 2016; Rafols et al., 2018).

#### 3.5.5 Implantation

Finally, a fifth method, less common, can also be used and consists in the implantation of nanoparticles inside the sample before performing the SALDI‐MSI analysis. This method is mainly developed in the group of Woods (Jackson et al., 2014; Muller et al., 2015, 2017; Roux et al., 2015). In this method, silver nanoparticles are implanted in a tissue section using an NPlanter (Ionwerks Inc), which produces Ag vapor (by magnetron sputtering) that condenses into 0.5–15 µm, pure, singly negatively charged silver nanoparticles within a gas‐filled condensation zone. The nanoparticles with the desired size (usually 6 nm) are then selected using a quadrupole mass filter. Next, the selected nanoparticles are accelerated, formed into a beam, focused on the sample and finally electrically rastered over the sample surface to ensure uniform implantation. The implantation of the nanoparticles offers high reproducibility and also eliminates the aggregation issue of colloidal nanoparticles. The spatial resolution is also very high as this dry method avoids the analytes to diffuse in the sample (Muller et al., 2017). However, it requires specialized instrumentation for the implantation, such as the NPlanter.

## 4 SELECTED APPLICATIONS OF SALDI‐MS IMAGING

**Figure 17:**
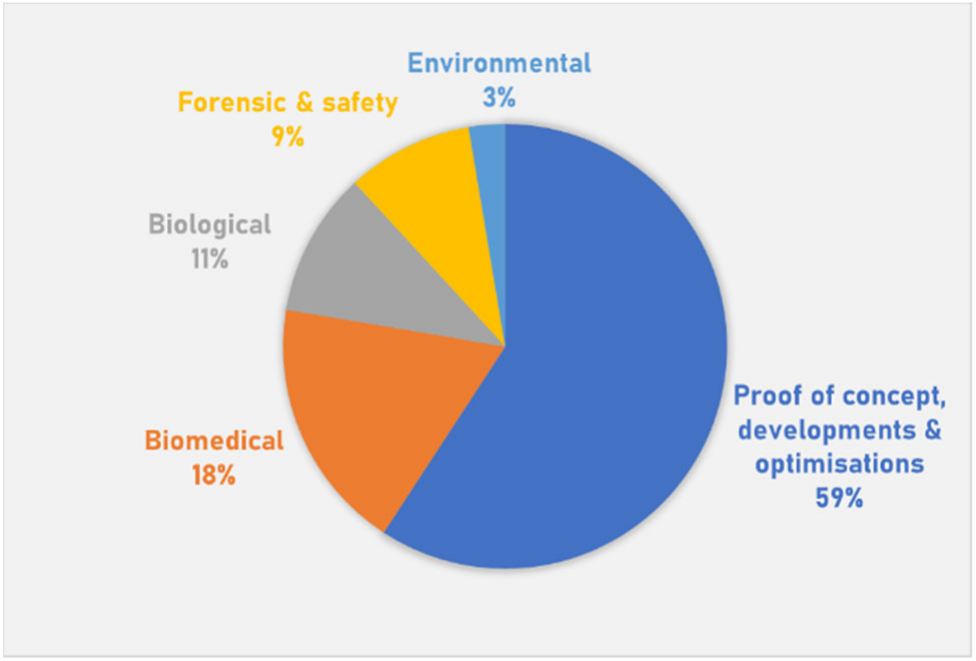
Fields of application of SALDI‐MS imaging[Color figure can be viewed at wileyonlinelibrary.com]

Most applications of SALDI nanosubstrates have focused on enzymatic assays, forensics, metabolite identification in biofluids or potential platforms for food analysis and only a few have been used for MSI (Lopez de Laorden et al., 2015). As previously presented in Figure 7, the samples already analyzed by SALDI‐MS imaging are relatively undiversified, with almost half of them belonging to murine tissues. Therefore, the applications of SALDI‐MSI are also quite limited up to these days but are expected to expand. Moreover, as the technique is still in its beginning, most publications are devoted to proofs of concept, in particular demonstrating the capabilities of novel nanosubstrates to image specific types of analytes, to the development of new aspects in SALDI‐MSI such as new substrate deposition/utilization method or to the optimization of the SALDI‐MSI technique, without a specific or applied context, as shown in Figure 17. In this section, we present some selected SALDI‐MSI applications in the biomedical, biological, environmental, and forensic fields.

### 4.1 Biomedical applications

As previously shown in Figure 17, the biomedical area has already been able to take advantage of SALDI‐MS imaging experiments, especially for the study of cancerous tissues, which are among the most common samples analyzed (Figure 7). In particular, different approaches based on SALDI‐MS imaging were developed to investigate the effect of chemotherapy or to discriminate normal and cancerous tissues. An early example was published by Tang et al. in 2011. The authors sputtered a 4‐nm thick layer of gold on 14‐µm thick fresh frozen mice cancerous tissues with the aim to discriminate the highly heterogeneous regions of the tumor tissue based on metabolic profiling obtained by SALDI‐MSI (Tang et al., 2011b). Some ions characterized by specific spatial distributions were used to differentiate the tumoral regions. For example, deprotonated taurine (*m/z* 124.0063), important for osmoregulation of tumors, was distributed all over the tumor tissue section. On the other hand, deprotonated adenine (*m/z* = 134.0473), a key component of ATP metabolism, was detected in most part of the tissue except in necrotic regions. In this study, the authors took profit of the sputtering method to coat homogenous Au layers on the tissue section, which limited the signal intensity fluctuations and thus improved the molecular images quality. The metal layers used as LDI‐assisting materials also made the sample surface conductive, which allowed the use of scanning electron microscopy to provide supplementary information on the tissue sections.

Metabolic biomarker discovery by SALDI‐MSI was also at the center of the work of Niziol and her colleagues who employed their gold nanoparticle enhanced target (AuNPET) as substrate to image renal cell carcinoma with the aim to differentiate between normal and cancerous tissues (Niziol et al., 2016). The tissues were used as received after surgery and imprinted on the AuNPET. Normal renal tissue and renal cell carcinoma were differentiated based on the presence of potential biomarkers, such as diglyceride DG(18:1/20:0) and protonated octadecanamide ions, both exhibiting high intensities in the cancerous areas of the tissue sample.

**Figure 18:**
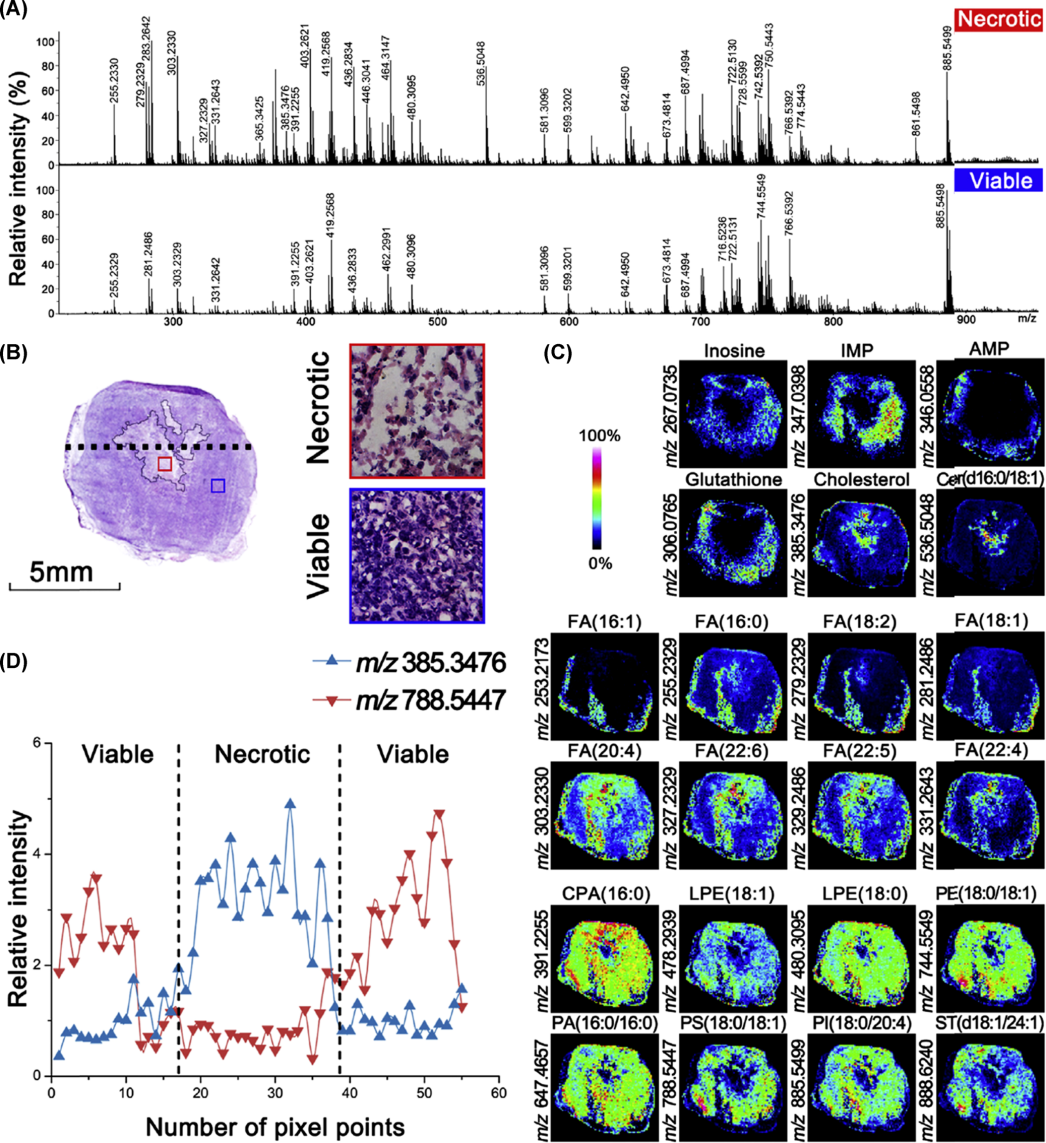
SALDI‐MSI of molecular heterogeneity in a mouse breast cancer tissue with graphene oxide as nanosubstrate, in negative ionization mode. (A) Representative mass spectra of the framed necrotic (red) and viable (blue) tumor regions in the tissue section. (B) Optical image of the H&amp;E‐stained adjacent tumor tissue section with necrotic tumor region outlined in the middle, and the framed areas of necrotic (red) and viable (blue) tumor regions. (C) SALDI‐MSI ion images of the whole tumor tissue section. Adenosine monophosphate (AMP), inosine monophosphate (IMP). (D) A plot of the relative intensity of the ions at*m*/z 385.3476 (cholesterol) and *m*/*z*788.5447 (phosphatidylserine) as a function of the location along the dotted black line marked in H&amp;E stained optical image. Reprinted with permission from Zhou et al. (2017). © 2017 Elsevier B.V [Color figure can be viewed at wileyonlinelibrary.com]

Zhou et al. also studied the molecular heterogeneity in tumor tissues based on the differences in small molecules profiles in the mass spectra of necrotic (red) and viable (blue) tumor regions, as shown in Figure 18A (Zhou et al., 2017). SALDI‐MSI was performed by coating graphene oxide (initially in water) on 12‐µm thick fresh frozen murine breast cancer tissue section. The SALDI‐MSI results (Figure 18C) are consistent with the H&E staining (Figure 18B), showing the heterogeneity in the whole tissue section. However, compared with H&E staining, SALDI‐MS images also highlighted molecular changes between the different regions of the tumor, as shown in Figure 18C. For instance, the peripherical viable tumor region is characterized by a predominant presence of glycerophospholipids (CPA, LPE, PE, PA, PS, and PI) and sulfatides (ST). On the contrary, ceramides (Cer) and cholesterol exhibit higher intensities in the necrotic tumor area compared with the viable region. Figure 18D clearly depicts the opposite changes in the expression of cholesterol (*m/z* = 385.3476) and phosphatidylserine (PS) (*m/z* = 788.5447) between the necrotic and peripherical viable tumor regions.

Several studies also employed SALDI‐MSI to monitor the decrease in the abundance of cancer biomarkers in response to chemotherapy and to analyze the distribution of anticancer drugs inside single cells or tissue sections. For example, O'Brien et al. monitored metabolic changes occurring in single cancerous cells as well as metabolic responses to chemotherapy in mouse breast tumor xenograft (O'Brien et al., 2013). Their study was based on the measure of the thymidine kinase (TK1) activity, which is a well‐established model for the evaluation of the proliferation potential of a tumor. TK1 activity was monitored via the 3’‐deoxy‐3’‐fluorothymidine (FLT) metabolism, in which FLT is taken up by the cells and phosphorylated into FLT monophosphate (FLT‐MP) by TK1. FLT is then transported out of the cells while FLT‐MP is retained. The accumulation of FLT‐MP in the cells can serve to detect a proliferating tumor. In the study, highly proliferative Raji Burkitt's lymphoma cells were first treated with rapamycin or FLT and then deposited on an etched silicon surface coated with bis(heptadecafluoro‐1,1,2,2,tetrahydrodecyl)tetramethyldisiloxane, used as nanosubstrate. The intracellular uptake of rapamycin and FLT was then detected as well as the FLT metabolism, which was monitored via the FLT‐MP/FLT intensity ratio. Second, mice were treated with docetaxel and FLT. FLT‐MP/FLT ratiometric images of 4‐µm thick sections were finally generated to evaluate the effect of the docetaxel treatment.

In an alternative approach, Morosi et al. employed SALDI‐MSI with TiO_2_ nanoparticles to measure the distribution of paclitaxel, an anticancer drug, inside fresh‐frozen normal and neoplastic solid tumor sections, and in relation to the dose administered (Morosi et al., 2013). Fresh‐frozen tissues were cryosectioned in 14‐µm thick sections and TiO_2_ nanoparticles were sprayed on these sections with an airbrush. Morosi et al. were able to visualize the different distributions of paclitaxel in normal and tumor tissues, related to the dosage schedules and pathological features of the tumors. The homogeneous deposition of the TiO_2_ nanoparticles on the tissue sections allowed to overcome the variability in signal response, which can be encountered in MALDI‐MSI due to heterogenous matrix/analytes co‐crystallization.

**Figure 19:**
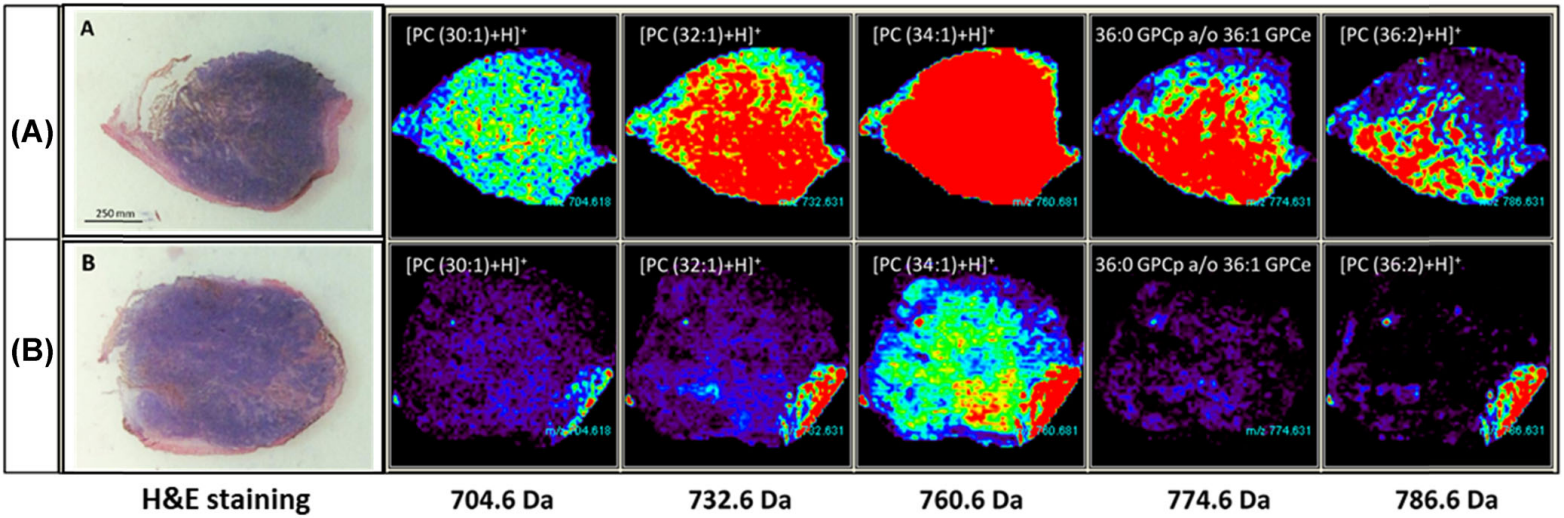
(A) Optical H&amp;E‐stained image and SALDI‐MS images of the control tumor tissue. (B) Optical H&amp;E‐stained image and SALDI‐MS images of the PHO‐S treated tumor tissue. Reprinted with permission from Tata et al. (2012). © 2012 American Chemical Society [Color figure can be viewed at wileyonlinelibrary.com]

On the other hand, Tata et al. studied the effect of cancer treatment with synthetic phosphoethanolamine (PHO‐S) based on characteristic lipid profiles of melanoma tumors (Tata et al., 2012). The control and PHO‐S‐treated 30‐µm thick melanoma tumor sections were imprinted on the surface of a NALDI™ target. First, Tata et al. attempted to identify potential lipid biomarkers by analyzing control and treated tumors. They found that phosphatidylcholines (PC, a class of phospholipids), among others, were good candidates. Then, the effect of PHO‐S treatment, inhibiting PC biosynthesis, was assessed using SALDI‐MSI. They demonstrated that PHO‐S treatment leads to a substantial reduction in the abundance of the phospholipid biomarkers, as shown in Figure 19. Two advantages of SALDI‐MSI were drawn from this study: (i) the NALDI target is selective for lipids, hence avoiding interference from other tissue components and (ii) the imprint on the NALDI target is characterized by a reduced salt content, thus eliminating the sodium and potassium adducts and their isobaric interferences. Overall, these two advantages led to the acquisition of simplified mass spectra.

Cancerous tissues were also already studied following a highly sensitive and selective “mass‐tag reporter” approach (R. Huang et al., 2015) as already discussed in Section 3.4.

Apart from cancer, other diseases have been studied by SALDI‐MSI, such as middle cerebral artery occlusion (Guan et al., 2018). In their work, Guan et al. used polyvinylpyrrolidone‐capped silver nanoparticles to investigate the variations in the lipid profiles between the healthy part of a rat brain and the one altered by infarctions. The brain tissue was fresh frozen and cryosectioned in 10‐µm thick sections onto which the nanoparticles were sprayed. Several metabolic changes between the normal and the ischemic regions were detected using SALDI‐MSI. For instance, K^+^ adducts of most phospholipids and sphingomyelins were significantly down regulated in the ischemic area, while their Na^+^ adducts were highly expressed. These results are relevant as the insufficient blood supply interrupts the ATP metabolism leading to a dysfunction of Na^+^/K^+^ ion gradient. Guan et al. also showed that most unsaturated fatty acids, prostaglandins, cyclic phosphatidic acids, vitamin A, neuraminic acid, and 5‐OH‐tryptophan were under expressed in the ischemic part of the brain, while saturated fatty acids, ceramides, hexanoylcarnitine and stearaldehyde were overexpressed. In Guan's study, SALDI‐MSI turns out to be a sensitive method to simultaneously analyze multiple classes of lipids, which is still challenging using conventional MALDI‐MSI.

In another study, Fincher et al. performed SALDI‐MSI to study human inflammatory skin disease (hidradenitis suppurativa) (Fincher et al., 2019b). In their work, they employed silicon nanopost arrays to image the distributions of neutral lipids, difficult to ionize with MALDI‐MSI. The human skin tissues were embedded in carboxymethyl cellulose, cryosectioned in 5‐µm thick sections, and finally deposited on the silicon nanosubstrate. The results of Fincher's study, based on the distributions of several neutral lipid species, suggest that the diseased tissues contain an increased bacterial load and open up new perspectives for the differentiation of healthy and diseased tissues. Additionally, the enhanced sensitivity in SALDI‐MSI for species hardly ionizable in MALDI‐MSI encourages the complementary use of the two LDI techniques.

Finally, Ag‐coated NIMS surfaces (i.e., fluorinated etched silicon surfaces) were used by Patti et al. in SALDI‐MSI to visualize the distribution of brain cholesterol metabolites in Smith‐Lemli‐Opitz syndrome, resulting from the mutation of the 7‐dehydrocholesterol reductase gene (Patti et al., 2010a). The patients suffering from Smith‐Lemli‐Opitz syndrome are thus unable to reduce 7‐dehydrocholesterol (7DHC) to form cholesterol, which leads to elevated levels of the 7DHC cholesterol precursor. In this study, OCT embedded frozen mouse brains were cryosectioned in 3‐5 µm thick sections, which were deposited on the nanosubstrate. The distribution of 7DHC and cholesterol were then imaged by SALDI‐MSI in diseased and healthy mouse brains. This study notably highlighted the increased 7DHC signals in the cerebellum and brainstem regions of the diseased brains compared with the healthy brains. NIMS substrates were highly valuable in this study as they allowed the imaging of sterol molecules such as cholesterol, which are notoriously challenging to detect with conventional MALDI‐MSI (Patti et al., 2010a).

### 4.2 Biological applications

Low molecular weight compounds also play essential roles in several biochemical pathways and fulfill important biological functions. SALDI‐MSI is an attractive tool to study the distribution of bioactive compounds in biological samples.

**Figure 20:**
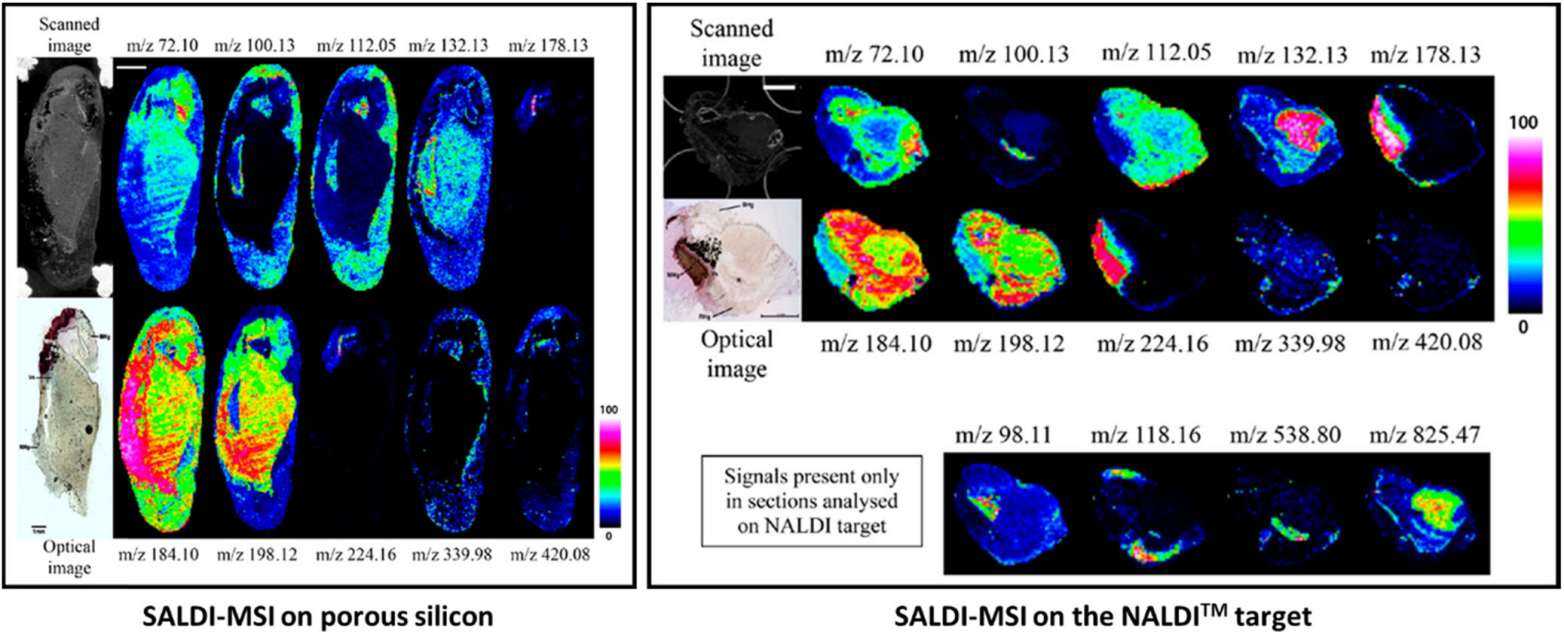
(Left) Ion intensity maps for the selected signals on a 30 µm thick section of the hypobranchial gland of a sea snail on porous silicon. The scanned image of the section before the removal from the surface and the brightfield microscopy image are also shown. (Right) Ion intensity maps for the selected signals on a 30 µm thick section of the hypobranchial gland of a sea snail on a NALDI™ target. The scanned image of the section before the removal from the NALDI target surface and the brightfield microscopy image are also shown. The bottom ion intensity maps show signals detected on the NALDI target but not present using porous silicon. Adapted with permission from Ronci et al. (2012). © 2012 American Chemical Society [Color figure can be viewed at wileyonlinelibrary.com]

For example, Ronci et al. interrogated the hypobranchial gland of a marine mollusc to investigate the distribution of biologically active brominated precursors of Tyrian purple (a natural dye), using SALDI‐MSI (Ronci et al., 2012). The point in studying the precursors of Tyrian purple is that they might induce apoptosis in cancer cells. However, the biosynthesis of Tyrian purple and its bioactive precursors remains unclear. In their study, hypobranchial glands were cryosectioned to 30‐µm thick sections and imprinted either on a porous silicon surface or on a NALDI™ target to compare the performance of the two types of nanosubstrates. The SALDI‐MS images are shown in Figure 20. The *m/z* 339.98 and *m/z* 420.08 signals are of particular interest as they are associated with compounds known to be synthesized in the hypobranchial gland, respectively the reduced form of the tyrindoxyl sulfate and the 6,6′‐dibromoindigo. As shown in Figure 20, some ions can also be used to localize histological regions. For instance, the ions at *m/z* 184.10 and 198.12 are mainly located in the rectal hypobranchial gland region. The ion at *m/z* 184.10 also appears in the rectum, dispersed via the vascular sinus. On the other hand, the ion at *m/z* 224.16 is concentrated around the medial region of the hypobranchial gland. The ion at *m/z* 72.10 was found in the vascular sinus adjacent to the branchial region, but not in the hypobranchial gland tissue. Some ions are linked with the mucus secreted by the gland, such as *m/z* 100.13 and *m/z* 112.05. In addition, this study shows the utility of several nanosubstrates and their complementarity. Indeed, as shown in Figure 20, some ions are detected on the NALDI™ target but not on the porous silicon, demonstrating the possibility to selectively extract different classes of compounds by changing the nanosubstrate surface chemistry and/or morphology. The ions only detected with the NALDI™ target are characterized by *m/z* values of 98.11 (associated with the rectal gland), 118.16 (linked to the rectal hypobranchial gland), 538.80 (associated with the rectum), and 825.47 (detected in the mantle tissue and in the vascular sinus).

Several years later, the same research group investigated the in situ temporal changes in the biodistribution of brominated indoles and choline esters at different stages of the reproductive cycle of muricid molluscs (Rudd et al., 2015). In this study, fresh‐frozen hypobranchial glands of the marine molluscs were cryosectioned (in 15‐µm thick sections) and imprinted on porous silicon substrates. In particular, this study highlighted the colocalization of two secondary metabolites (i.e., murexine and tyrindoxyl sulfate) in the hypobranchial glands of the marine molluscs and the transfer of murexine to the capsule gland, and then to the egg capsules, where chemical ripening results in Tyrian purple formation (Rudd et al., 2015).

Another example, using SALDI‐MSI to image low molecular weight metabolites in garlic, was published by Misiorek et al. (2017). In this study, a garlic clove cross‐section was imprinted on a gold nanoparticle enhanced target (AuNPET). Organosulfur compounds, which are the most important group of garlic compounds with bioactive properties, were detected in the imprint. These compounds include alliin/isoalliin (H^+^ adduct at *m/z* 216), diallyl sulfide (H^+^ adduct at *m/z* 115), allyl mercaptan (K^+^ adduct at *m/z* 112) and allyl methyl tetrasulphide (K^+^ adduct at *m/z* 222). Organoselenium compounds, including selenomethionine (K^+^ adduct at *m/z* 235) and methaneseleninic acid (K^+^ adduct at *m/z* 188) were also found in the garlic imprint. The distributions of fatty acids, amino acids, and dipeptides were also visualized.

In a similar way, Niziol et al. used ^109^Ag nanoparticle enhanced target (^109^AgNPET) to image low molecular weight compounds in strawberry and correlate their spatial distribution with their biological function (Niziol et al., 2019). The strawberry cross‐section was imprinted on a ^109^AgNPET substrate. The authors were able to image the distribution of over thirty metabolites present in strawberries and divided into two main groups: primary (amino acids and sugars) and secondary metabolites. Different kinds of compounds were identified and categorized into flavor compounds, phenols, vitamins, sugars, amino acids, carboxylic acids, or flavonoids. In particular, Niziol et al. showed that the distributions of these compounds are not homogeneous and are related to the biological function of the metabolite in the strawberry. For example, γ‐aminobutyric acid (Na^+^ adduct at *m/z* 126.0626), quinic acid, (K^+^ adduct at *m/z* 300.9676), vitamin C (^109^Ag^+^ adduct at *m/z* 284.9363), and catechin (H^+^ adduct at *m*/*z* 706.1892) are mostly located under the skin, which is due to their protective function. On the other hand, flavor compounds such as aldehydes (hexanal [H^+^ adduct at *m/z* 101.0961], benzaldehyde [K^+^ adduct at *m/z* 145.0050]) and ketones (1‐penten‐3‐one [H^+^ adduct at *m/z* 85.0648], geranylacetone [Na^+^ adduct at *m/z* 217.1563]) were found throughout the strawberry flesh, both in the inner core and in the cortex layer. Some other compounds such as asparagine (H^+^ adduct at *m/z* 133.0608), lysine (H^+^ adduct at *m/z* 147.1126), gambiriin C (^109^Ag^+^ adduct at 671.0617), oxalic acid (Na^+^ adduct at *m/z* 112.9045), and 2‐methylbutanoic acid (Na^+^ adduct at *m/z* 148.0604) were found on or around the surface of the achenes. Their localization is probably linked with the sites of biosynthesis of these compounds located in chloroplasts.

Microbial interactions were also already imaged by SALDI‐MSI. For example, Chen et al. studied the metabolic interactions between two fungal strains producing a dense mass of aerial mycelia, namely *Phellinus noxius* and *Aspergillus*, which are difficult to image using traditional MSI methods (Chen et al., 2018). The fungal culture was imprinted on a nanostructured silicon surface. The aim of the study was to discover antifungal agents from *Aspergillus* displaying an inhibitory effect on the cocultured aggressive fungal pathogen *P. noxius*, which causes the brown root rod disease.

**Figure 21:**
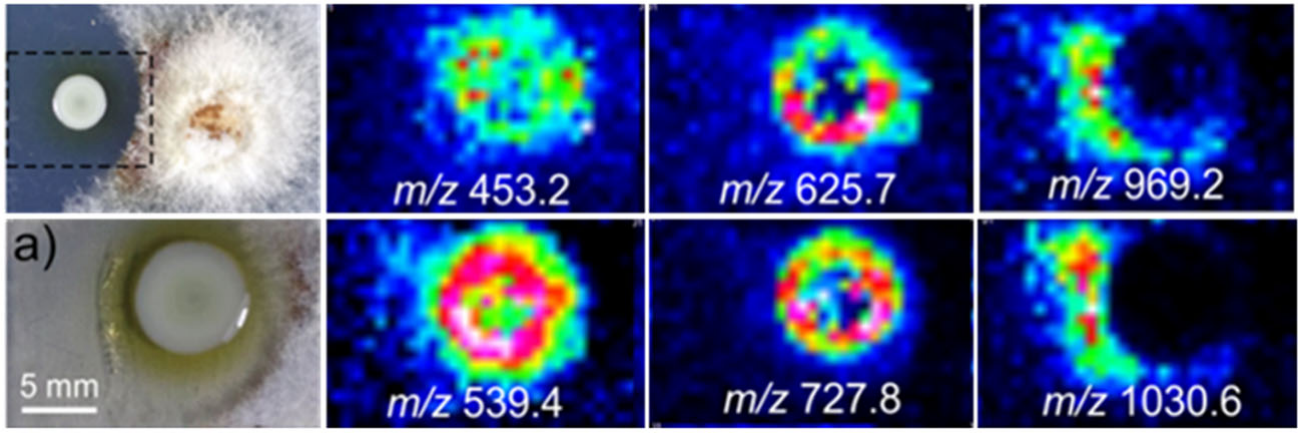
Application of the TiO_2_nanowire substrate for SALDI‐MSI of the microbial coculture of*Burkholderia cenocepacia 896T2*versus Phellinus noxius. The black dashed rectangle represents the SALDI‐MS image area containing the bacteria. Reprinted from Dutkiewicz et al. (2019) (CC BY‐NC‐ND 4.0) [Color figure can be viewed at wileyonlinelibrary.com]

*P. noxius* was also studied in the work of Dutkiewicz et al. in which trichloro (1H, 1H, 2H, 2H‐perfluorooctyl)silane functionalized TiO_2_ nanowires were used as solid nanosubstrate to image the distribution of the secondary metabolites in the microbial coculture involving bacteria (*Burkholderia cenocepacia 869T2*) and fungi (*P. noxius*) (Dutkiewicz et al., 2019). *B. cenocepacia 869T2* is a bacterium of particular interest as it is capable of inhibiting several phytopathogens such as *P. noxius*. Bacteria‐related metabolites produced in reaction to the presence of the fungi were observed in SALDI‐MSI, whereas they were not revealed by MALDI‐MSI. In particular, the [M+Na]^+^ signals at *m/z* 453.2, 539.4, 625.7 and [M+K]^+^ signal at *m/z* 727.8 (Figure 21) correspond to poly‐(R)‐3‐hydroxybutyrate polymers. The poly‐(R)‐3‐hydroxybutyrate polymers serve primarily as an energy source but also enhance the resistance of the bacteria to various stress conditions. Moreover, the spatial distribution of the ions at *m/z* 969.2 and 1030.6 indicate that the fungi interfered with the bacteria in the co‐cultural conditions, as shown in Figure 21. This study highlighted the improvement of the selectivity for specific analytes due to chemical modifications of the nanosubstrate as well as the use of the same nanosubstrate in both ionization modes (positive and negative).

In a different context, Ferreira et al. reported the direct lipid characterization at the single‐cell level by SALDI‐MS imaging for the discrimination of embryos compartments (Ferreira et al., 2014). Embryos were fixed with 4.0% paraformaldehyde solution and then deposited on a TLC silica plate, acting as SALDI nanosubstrate. The SALDI‐MSI approach allowed the identification of biomarkers allowing the differentiation of blastomere and intact zona pellucida, without any sample preparation. For example, the zona pellucida is characterized by the presence of lower molecular weight lipids, such as phosphatidylethanolamines [PE(24:0/20:0) + H]^+^ (*m/z* 861) and phosphatidic acid derivatives [PA(20:0/20:3) + H]^+^ (*m/z* 657). On the contrary, higher molecular weight lipids are found in blastomeres, such as phosphatidylethanolamines [PE(16:0/18:1)‐15‐isoLG pyrrole + K]^+^ (*m/z* 1073) and ceramides [Cer(18:1/22:0) + Na]^+^ (*m/z* 1277).

### 4.3 Environmental applications

Environmental studies are at the center of modern research challenges. In this context, SALDI‐MSI can be highly valuable to image the distribution of pollutants or pesticides.

**Figure 22:**
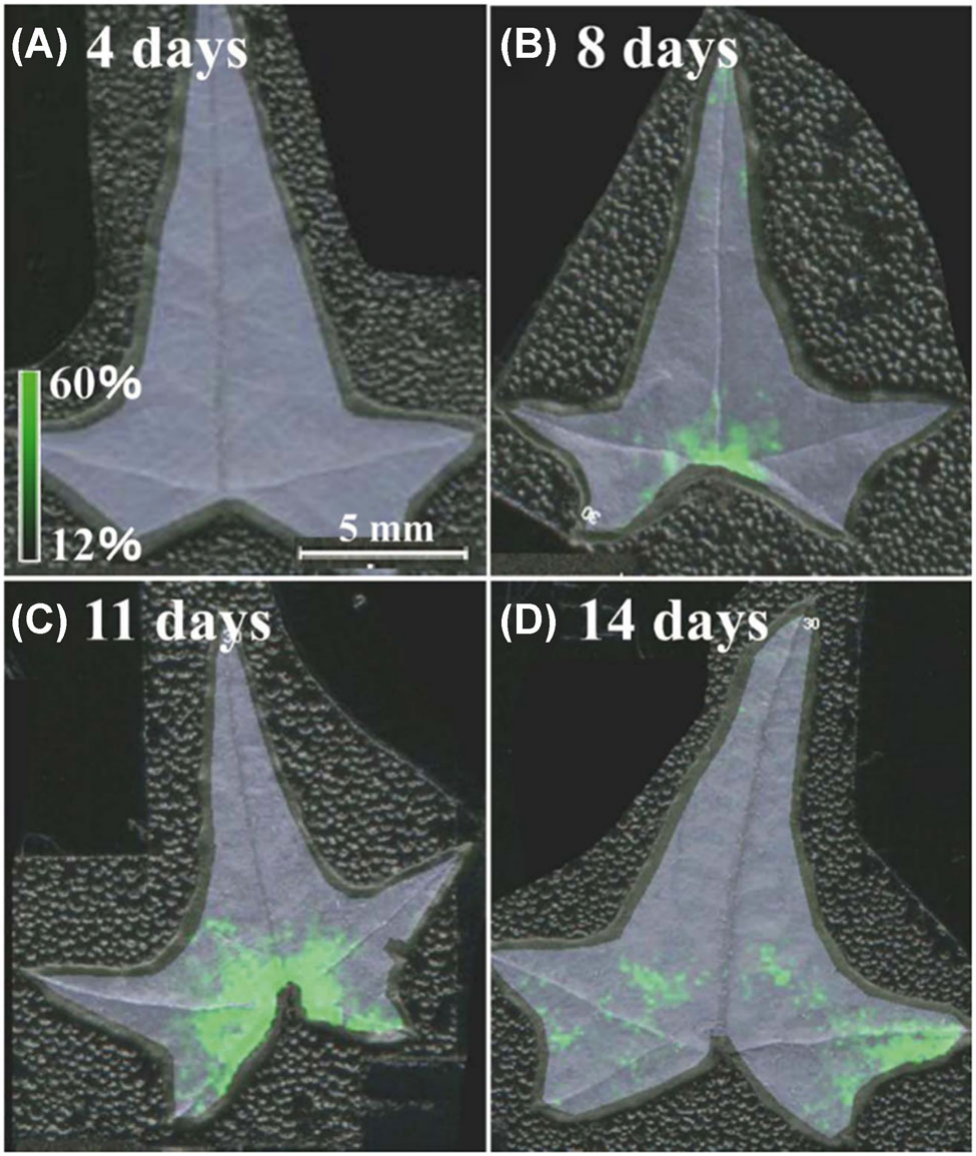
SALDI‐MS images of acephate sodium adduct ion at m/z 206 in Ivy leaves (Hedera) containing a commercial pesticide after (A) 4, (B) 8, (C) 11, and (D) 14 days. Reprinted with permission from Ozawa et al. (2016). © 2016 The Japan Society for Analytical Chemistry [Color figure can be viewed at wileyonlinelibrary.com]

For example, Ozawa et al. demonstrated the use of SALDI‐MSI to monitor the distribution of an insecticide and its migration over time in plant leaves, as shown in Figure 22 (Ozawa et al., 2016). A horticultural chemical agent containing acephate as a vermicide was mixed into the soil in which the plant was grown. The plant leaves were then collected 4, 8, 11, and 14 days after the administration of the insecticide and stuck on a glass slide with double‐sided tape. A 10‐nm thick platinum film was finally sputtered on the leaves and SALDI‐MSI was performed following the signal of the acephate sodium adduct ion at *m/z* 206. Interestingly, these ions can be analyzed using SALDI‐MSI but they were not adequately detected by MALDI‐MSI due to the charge‐up effect, that is, the accumulation of charges that cannot be released, on nonconductive sample surfaces (Ozawa et al., 2016). The movement of the insecticide (which was initially absorbed by the roots and distributed into the plant) was eventually monitored into the leaves, as shown in Figure 22.

Niziol et al. also studied agricultural chemicals in plant materials (Niziol & Ruman, 2013b). *Mentha piperita* stem was collected from a plant polluted with 2‐methyl‐4‐chlorophenoxyacetic acid (MPCA), a potent and selective herbicide. The stem cross‐section was then imprinted on ^109^AgNPET to localize the herbicide inside the plant stem by SALDI‐MSI. The MCPA was found as a sodium adduct at *m/z* 223.014 mainly in the outer parts of stem cross‐section. Other ions such as a metabolite of the herbicide 5‐methylthiopentanaldoxime K (potassium adduct at *m/z* 186.035) were also found in the stem imprint.

### 4.4 Forensic applications

The SALDI‐MSI capability to image small molecules also led to its rapidly growing popularity in the forensic field. In this area, SALDI‐MSI has a great potential, for instance, to visualize the distribution of illicit drugs and their metabolites, to spot counterfeits of banknotes, checks and other questioned documents and to investigate the molecular composition of latent fingerprints, even on nonconductive surfaces such as paper.

**Figure 23:**
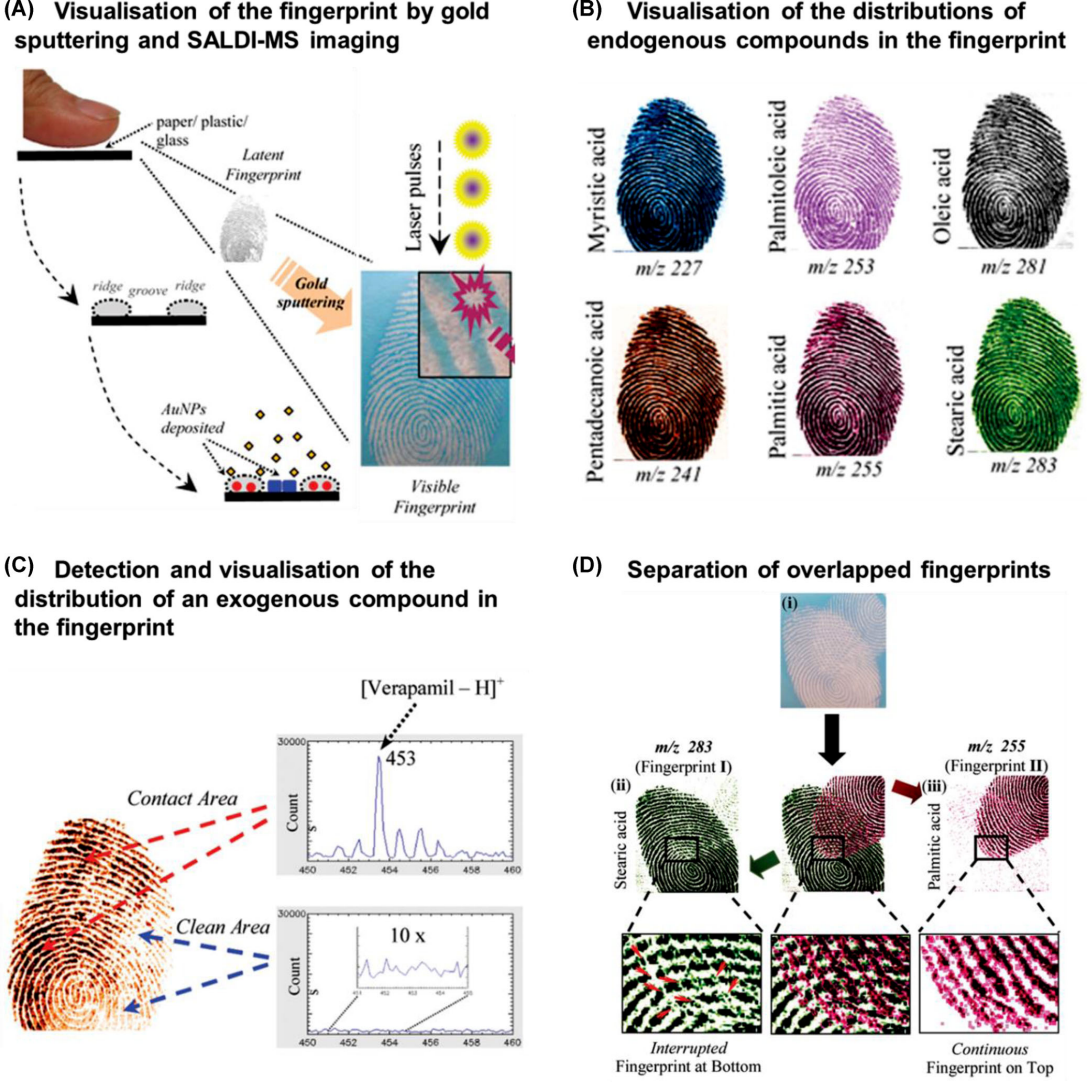
(A) Schematic diagram showing the visualization of the fingerprint using gold sputtering and the analysis of the fingerprint by SALDI‐MSI (B) Molecular SALDI‐MS imaging of fingerprint based on the spatial distributions of different endogenous compounds (C) Molecular SALDI‐MS imaging of an exogenous compound (verapamil) embedded in a fingerprint (D) Separation of the physical domains of two overlapped fingerprints based on their respective molecular images. Adapted with permission from Tang et al. (2010). © 2010 American Chemical Society [Color figure can be viewed at wileyonlinelibrary.com]

As previously indicated in Figure 7, fingerprint analysis is a large area of the SALDI‐MSI research since it is in the top three kinds of analyzed samples, after murine tissues and plant samples. Several authors used SALDI‐MSI for the investigation of latent fingermarks, which is one of the most important and most common tasks in forensic science, allowing biometric identification. For instance, Tang et al. employed gold sputtering for both visualizing and analyzing the molecular composition of latent fingerprints by SALDI‐MSI (Figure 23A) (Tang et al., 2010). They imaged the distributions of endogenous (Figure 23B) and exogenous compounds (Figure 23C) embedded in the fingerprints and were also able to separate overlapped fingerprints (Figure 23C), demonstrating the capabilities of SALDI‐MSI in forensic investigations. Fingerprints were first imprinted on a support (e.g., glass coverslip, plastic film, white paper) and then visualized by gold sputtering. Indeed, gold is deposited on the ridges and grooves of the fingerprints in two different forms, exhibiting contrasting colors (i.e., pink on ridges and blue on grooves), as shown in Figure 23A allowing the visual observation of the fingerprints with naked eyes. Moreover, the sample surface became conductive after gold sputtering, and thus suitable for scanning electron microscopy, providing further microscopic scale information of the fingerprints. Finally, through SALDI‐MSI, chemical information was obtained from the fingerprint, providing additional information relevant to the individual identity and for the detection of hazardous or illicit substances. In particular, Tang et al. imaged the distribution of a trace amount of verapamil often used as a drug against hypertensive condition, embedded in the fingermarks, as shown in Figure 23C.

Gold‐based nanosubstrates were also employed by Sekula et al. to image low molecular weight compounds in fingerprints imprinted on AuNPET (Sekula et al., 2015b). Various compounds were detected such as inorganic salts (e.g., NaCl, KCl), simple organics (e.g., amino acids and short carboxylic acids), fatty acids, lipids, and a few detergents.

Lauzon et al. also interrogated latent fingerprints to determine their molecular composition using SALDI‐MSI (Lauzon et al., 2015). The fingerprints were imprinted either on ITO‐coated slides or on nonconductive paper sheets. Silver sputtering was then applied on top of the fingermarks and led to the deposition of a 14 ± 2 nm thick layer of silver. SALDI‐MS imaging analysis finally allowed the detection and imaging of numerous endogenous compounds, mainly in the form of [M+Ag]^+^ ions, such as cholesterol, squalene, wax esters, diglycerides, triglycerides and fatty acids, as well as other exogenous substances, including ditallowdimethylammonium ions and polyethylene glycol, originating from personal care and domestic products. Furthermore, odd carbon number fatty acids, probably coming from skin bacteria, were also detected and localized in the fingerprint, opening new opportunities in the detection of biological agents carried or manipulated by suspects.

Guinan and her colleagues also studied fingerprints imprinted on nanostructured silicon (Guinan et al., 2015a, 2015b). In a first study, the fingerprints were imprinted on a porous silicon surface functionalized with (pentafluorophenyl)propylchlorodimethyl silane (Guinan et al., 2015c). SALDI‐MSI was then used for the direct detection of endogenous lipids (e.g., cholesterol) as well as drugs and their metabolites. For instance, the analysis of the fingerprint from a smoker with clean hands highlighted the presence of nicotine (*m/z* 163), which was not detected in the fingerprint from non‐smokers. The authors also demonstrated the capability of SALDI‐MSI to highlight the skin contact with illicit drugs (e.g., methamphetamine) of a person who has handled them. Interestingly, Guinan et al. were also able to evaluate drug consumption and to confirm the secretion of the drug into the fingerprint sweat as opposed to the contamination of the skin through drug handling. In their study, the fingerprint of an individual enrolled in a heroin replacement program was analyzed and highlighted the presence of methadone (*m/z* 310) whereas heroin was not detected. Furthermore, 2‐ethylidene‐1,5‐dimethyl‐3,3‐diphenylpyrrolidine (*m/z* 278), a common metabolite of methadone, was also detected in the fingerprint, confirming the drug consumption. In their second study, Guinan et al. employed porous silicon silanized with (tridecafluoro‐1,1,2,2‐ tetrahydrooctyl)dimethylchlorosilane and further coated by a layer of sputtered silver (Guinan et al., 2015a). Fingerprints were imprinted on the Ag sputter‐coated functionalized porous silicon surface. Several endogenous compounds were detected such as fatty acids (palmitic *m/z* 363.15, linoleic *m/z* 387.145, oleic *m/z* 389.160, and stearic *m/z* 391.176), cholesterol (*m/z* 493.259), wax esters (30:1 *m/z* 557.348, 32:1 *m/z* 585.380, 34:1 *m/z* 613.411, 36:1 *m/z* 641.442 and 38:1 *m/z* 669.473), and triacylglycerols (45:1 *m/z* 785.663 and 48:1 *m/z* 827.710). Exogenous compounds were also found in the fingerprint, including benzyldimethyldodecylammonium (*m/z* 304.300), behentrimonium (*m/z* 368.425), and dimethyldioctadecylammonium (*m/z* 550.629), commonly found in household and personal care products.

Finally, NALDI™ plates were also used as nanosubstrates for the analysis of illicit drugs in fingerprints by SALDI‐MSI (Skriba & Havlicek, 2018). In Skriba's study, methamphetamine, heroin, and cocaine distributions were visualized in latent fingerprints imprinted on the NALDI™ plate (Skriba & Havlicek, 2018).

Another part of forensic science focuses on document analysis and counterfeiting. As previously presented in Section 3.4, Creran et al. used SALDI‐MSI in anticounterfeiting applications (Creran et al., 2012). As a reminder, they functionalized gold nanoparticles with surface ligands characterized by unique MS signatures and patterned these functionalized nanoparticles onto a surface, providing discernible design through SALDI‐MSI, as previously shown in Figure 15.

**Figure 24:**
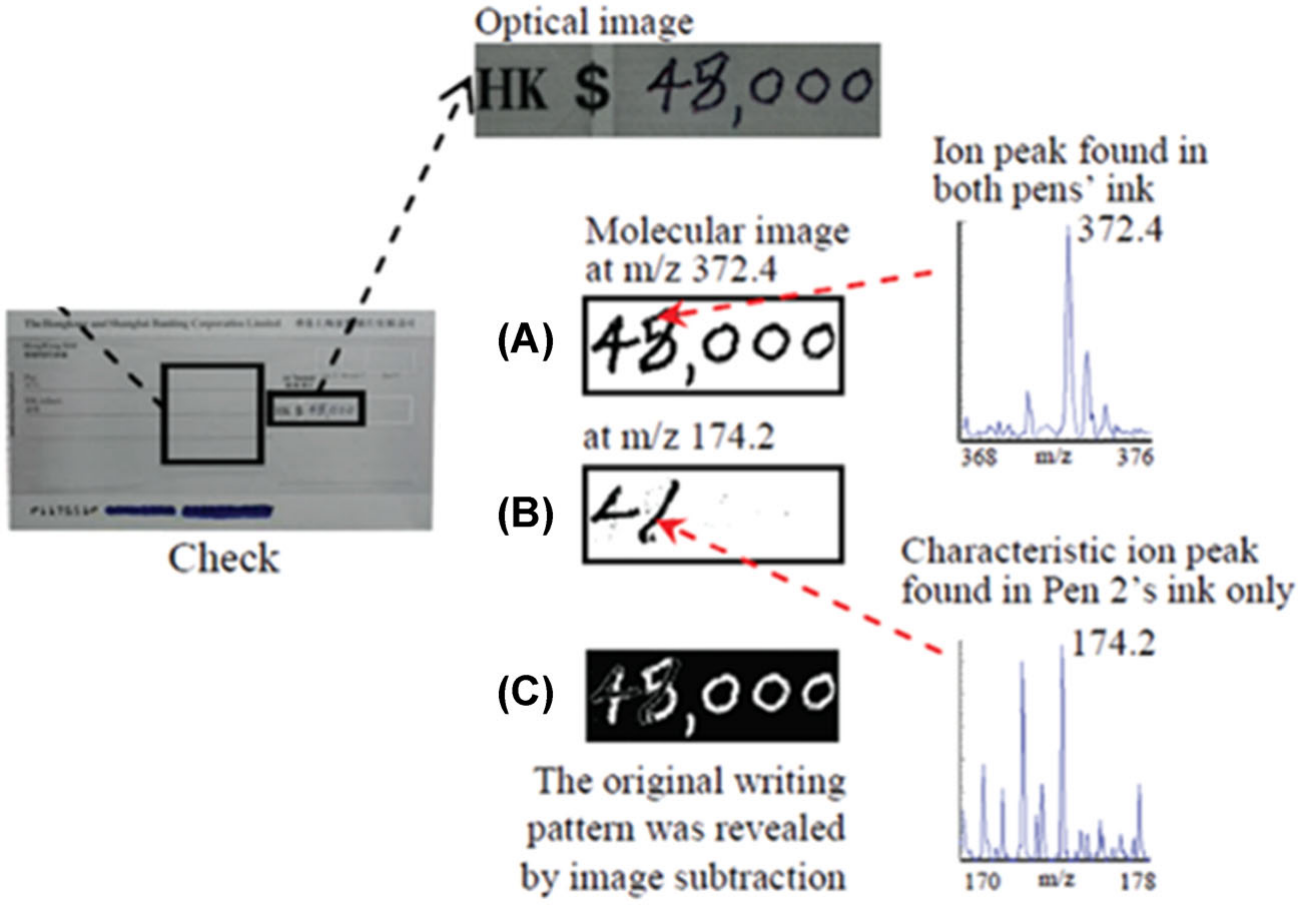
Mass spectrometry imaging analysis of a check. Identification of forged parts in altered writing. (A) Molecular image of crystal violet at*m*/*z*372.4, a common dye found in pen ink, matched the whole handwriting pattern. (B) Molecular image of a characteristic ion at*m*/*z*174.2, which was detected only in parts of the writing, suggested that the writing was written using more than one pen. (C) Original writing was revealed by subtracting the forged parts using an image processing program, ImageJ (NIH). Reprinted with permission from Tang et al. (2011a). © 2011 American Chemical Society [Color figure can be viewed at wileyonlinelibrary.com]

Tang et al. demonstrated that a solvent‐free SALDI‐MSI approach based on gold sputtering enables the detection and imaging of inks and visible and/or fluorescent dyes printed on banknotes or written on questioned documents (Tang et al., 2011a). First, they were able to detect the compounds found in visible and fluorescent inks printed on banknotes and image their distributions. The use of several inks printed in complex patterns is one of the important anticounterfeit features of banknotes and official documents. Then, by inspecting the overlapped regions of the molecular images, the authors were able to retrieve the ink printing order, which is also a useful security detail, in addition to the chemical composition and the printing patterns of the inks. Tang et al. also managed to detect check forgery by SALDI‐MSI, as shown in Figure 24. Writing inks of the same color may have different compositions as these inks are composed of a complex mixture of solvent, dyes, pigments and other additives. Based on the chemical composition of the ink, provided by SALDI‐MSI, the authors revealed the forged part of the altered numbers. Indeed, a characteristic ion at *m/z* 174.2 (Figure 24B) is detected in an uninterrupted way in the forged part of the check, implying that these patterns were written on top of the line originally written. By subtracting the molecular image of the number “48,000” by the molecular image of the forged part, Tang et al. revealed the original number “15,000” written on the check, as depicted in Figure 24C.

## 5 CONCLUSIVE REMARKS AND PERSPECTIVES

MSI, allowing the investigation of the spatially resolved distributions of many analytes, in a great variety of complex solid samples, at the molecular level, has become one of the most important and promising implementation of MS, providing unequaled insights, highly valuable in various scientific disciplines. In this context, MALDI‐MSI is generally viewed as the MSI reference technique, however the emergent SALDI‐MSI is a powerful alternative, especially for detecting small molecular species with low background interference. First introduced by Tanaka et al. in 1988, SALDI‐MS recently expanded thanks to the rapid development of nanomaterials. Actually, SALDI‐MSI and MALDI‐MSI are complementary techniques, both in terms of *m/z* range covered and analyzable compounds, and can be performed with similar instrumentation. Nevertheless, SALDI‐MSI offers many advantages compared with MALDI‐MSI but still suffers from some limitations that slow down its development.

### 5.1 Advantages and limitations of SALDI‐MS imaging

The implementation of nanostructured substrates in SALDI‐MSI is responsible for the main advantages of the technique, including the ability to visualize the distributions of small molecules, the improved selectivity toward specific analytes thanks to the natural affinity and/or the functionalization of the nanosubstrate, the signal amplification through the monitoring of mass “barcodes,” the analysis of nonconductive surfaces and the higher reproducibility (shot‐to‐shot and sample‐to‐sample) compared with conventional MALDI‐MSI.

However, although the improved performance achieved with nanostructured substrates in SALDI‐MSI has been increasingly recognized, SALDI‐MSI is still far less used than traditional MALDI‐MSI (Phan et al., 2016). Some limitations of the SALDI technique still hinder its expansion. First, the key principles of the SALDI fundamental mechanisms remain not fully understood and represent one of the most controversial part of the SALDI research (Law & Larkin, 2011), hindering its development and application (Zhu et al., 2020). Second, the lack of commercial solid nanosubstrates to perform reproducible SALDI‐MSI can be a barrier to the development of this technique. Moreover, the high costs of some nanomaterials, the difficulty to maintain stable colloids, and possible contamination of the mass spectrometers may also hinder the SALDI‐MSI development (Kim et al., 2011). Therefore, there is still much room for improvement in SALDI‐MSI and future developments are expected in the forthcoming years.

### 5.2 Commercial SALDI nanosubstrates

The current limited number of commercially available nanosubstrates for SALDI‐MSI may slow down its expansion. Three main types of nanosubstrates can be used in SALDI‐MSI: sputtered metals, solid nanostructured surfaces, and colloidal nanomaterials.

While interest in nanoparticles is growing because they share ideal features for MSI, the achievement of stable and reproducible preparations in research laboratories is still too often uncertain, even when the standard operating procedure is strictly carried out to the letter (Yang et al., 2020). However, more and more companies are selling nanoparticles of different compositions and sizes with the required reproducibility.

On the other hand, nanostructured solid substrates are generally preferred because they do not require any spraying procedure onto the sample, thus avoiding the analyte migration and also because they do not require any stabilizing agent, which may induce interference in the low *m/z* range. However, the manufacture of these solid nanosubstrates requires skills and also fit‐for‐purpose equipment that not all laboratories have. Moreover, only very few nanosubstrates are currently commercially available, including MassPrep DIOS™ target from Waters, NALDI™ target from Bruker Daltonics and REDIchip™ from Protea. Furthermore, these commercial solid nanosubstrates are manufactured as a 96‐spot arrays, designed for droplet analysis and not for whole tissue section imaging, considerably hindering the development of SALDI‐MS imaging.

### 5.3 SALDI fundamental mechanisms

By now, we still have only a limited understanding of the SALDI‐MS desorption/ionization fundamental mechanisms. However, the understanding of the processes occurring in SALDI‐MS as well as their correlation with the nanosubstrate properties would benefit both fundamental studies and applications (Law, 2010). To this end, further experiments are needed to gain additional insights into the key mechanisms involved in SALDI and to establish the role of the contributing properties (e.g., surface chemistry, morphology, etc.) of the nanosubstrate. The comprehension of the SALDI mechanisms is also necessary to optimize the parameters of the MS experiment. For example, the fragmentation process has to be controlled as it strongly influences the SALDI‐MS performance in terms of background signal interference and efficiency of the energy transfer from the laser to the analytes.

### 5.4 SALDI‐MSI instrumentation

While a major effort has been made upstream of the SALDI‐MSI analysis (sample preparation, fabrication of nanosubstrates, functionalization, deposition, etc.), very few, if any, developments have been made in the field of instrumentation. However, SALDI‐MS imaging offers new perspectives that would require an upgrade of the instrumentation. For example, the high spatial resolution offered by SALDI‐MSI cannot be fully exploited if the laser spot size and/or the moving stage displacement capabilities are limited to 10 µm. Additionally, since nanosubstrates are not or only slightly dependent on the irradiation wavelength, the use of tunable lasers in SALDI could possibly open up new horizons. Moreover, the use of solid nanosubstrates is not always easy due to problems of laser focus on the sample. An easier focusing of the laser on any type of support, of variable dimensions, would also be welcome.

### 5.5 Imaging of hardly ionizable compounds

The distributions of a wide range of molecular species have already been effectively visualized thanks to MSI. However, some analytes are still difficult to detect by conventional MALDI‐MSI due to low ionization efficiency, low abundance and low solubility of the analytes (Cramer 2016). This is the case of neutral carbohydrates, for example, which are hardly ionizable compounds (Patti et al., 2010b; Picca et al., 2017) due to their low proton affinity (due to the absence of basic or acidic group in the structure), their low or even non‐volatility and their thermolability (Wu et al., 2013). Yet, carbohydrates are of significant biological relevance as they are involved in various biological processes such as cell‐cell recognition, protein targeting, and metabolic diseases (Huang & Chang, 2012). Therefore, efficient and convenient MSI approaches for the analyses of native carbohydrates are urgently needed (Wu et al., 2013). The improvement of the specificity and the sensitivity of the MS analysis are also required. Chemical derivatizations may improve the sensitivity of the analysis but, in practice, for MSI, such modifications are unsuitable because of the complexity of the molecular mixtures present in the sample (Wu et al., 2013) and, on the other hand, because they involve an additional chemical preparation step, which adds a significant degree of complexity and preparation time. Chemical derivatizations may also induce additional variability in the analytical approach such as the delocalization of the small metabolites, affecting the spatial resolution. In contrast, SALDI‐MSI appears to be a suitable technique for the imaging of neutral carbohydrates as it does not require any derivatization (Patti et al., 2010b) or digestion and a more efficient ionization process is usually observed with SALDI‐MS (Fu et al., 2015).

SALDI‐MSI could also be valuable in the analysis of high molecular weight polymers, which are still difficult to analyze with conventional MALDI‐MSI. Most nanosubstrates exhibit strong photocatalytic activity. Upon UV laser irradiation, the nanosubstrates may therefore cause the degradation of high molecular weight polymers, generating small fragment ions, analyzable by SALDI‐MSI (Watanabe et al., 2008).

### 5.6 Quantitative SALDI‐MS imaging

While the field of MSI has seen a significant growth in recent years, absolute quantitative analysis by MSI still poses a real challenge (Ellis et al., 2014). The difficulty in extracting quantitative information from MSI is largely due to the high dependency of the MS signal on both the type of analyte and the local composition of the surface (Ellis et al., 2014), which may cause ion suppression and affect analyte desorption/ionization efficiency and ion stability that, in turn, may have a significant impact on the measured ion intensities (Gessel et al., 2014; Trim & Snel, 2016). It is even more dramatic in the case of MSI, where the chemical microenvironments of adjacent areas within the same sample section may be chemically and/or morphologically totally different (Gessel et al., 2014; Wu et al., 2020). This sample heterogeneity may, in some cases, result in varying desorption/ionization efficiencies across a single sample section (Gessel et al., 2014). In these cases, the measured ion intensities are not simply dependent on the surface concentration of the analytes (Ellis et al., 2014). In MALDI‐MSI, further complications arise from the use of an organic matrix. Indeed, the heterogeneity of the analyte/matrix co‐crystallization creating “sweet spots” (with corresponding problems of poor mass accuracy and poor shot‐to‐shot and sample‐to‐sample reproducibility [Chiang et al., 2010]) is a major factor preventing the quantitative analyses (Qiao & Liu, 2010). However, with experimental optimization and appropriate internal standard (Wall et al., 2004), SALDI‐MS has been proven to be capable of performing quantitative analyses (Go et al., 2003; Okuno et al., 2005; Wall et al., 2004), therefore opening new opportunities in quantitative MSI (Cazier et al., 2020; Wu et al., 2020). In particular, ion signal calibration and normalization strategies, adapted to each specific microenvironment of the sample, are required (Wu et al., 2020). It is worth to note that quantitative methods are particularly required especially for the understanding of biological process (Wu et al., 2020) and to quantitate small pharmaceuticals in tissues, for which SALDI‐MSI represents a technique of choice.

### 5.7 Multidimensionality and multimodality

While MSI is a widespread and well‐established technique, it also suffers from limitations, for example, in lacking of ionization yield for some families of analytes (e.g., synthetic polymers such as polyethylene), by its limited spatial resolution compared with other imaging modalities (Buchberger et al., 2018) or by its impossibility to differentiate structural isomers. To overcome these limitations and to maximize the molecular information that can be extracted from the samples, MSI can be combined with other complementary analytical techniques.

For instance, a currently booming and promising coupling is the combination of ion mobility and MSI (Djambazova et al., 2020; Neumann et al., 2020; Mesa Sanchez et al., 2020; Sans et al., 2018; Spraggins et al., 2019; Soltwisch et al., 2020). This combination is particularly interesting for the study of biological samples, which present complex chemical composition and morphology (Sans et al., 2018). Indeed, MSI cannot separate structural isomers, which hampers the analysis of small metabolites and lipids, characterized by structural complexity and abundance of isomers. Coupling ion mobility with MSI allows the separation of isobaric and isomeric molecular species (Djambazova et al., 2020; Sans et al., 2018). Several preliminary examples have already demonstrated the combination of ion mobility and SALDI‐MS (Adamov et al., 2011; Kuzishchin et al., 2015; Tempez et al., 2005; Ugarov et al., 2004).

MSI can also be combined with complementary imaging modalities having different analytical assets (Bodzon‐Kulakowska & Suder, 2016; Ho et al., 2017; Siegel et al., 2018), such as microscopy (Van de Plas et al., 2015), Raman spectroscopy (Ahlf et al., 2014; Bocklitz et al., 2013), infrared spectroscopy (Neumann et al., 2018; Niziol et al., 2020), fluorescence (Jones et al., 2020; Si et al., 2016) or other MSI techniques (De San Roman et al., 2018; Eijkel et al., 2009; Fincher et al., 2020a). Multimodal approaches, providing a comprehensive analysis that could not be achievable with a single imaging technique, are currently rapidly expanding and represent a promising avenue in many disciplines. Furthermore, the SALDI nanosubstrates can be used for a panel of analytical techniques (Abdelhamid, 2018), providing adapted substrates for multimodal imaging. For instance, various nanomaterials have already been used for combined Surface‐Enhanced Raman Scattering (SERS) and SALDI‐MS analysis (Alessandri et al., 2016; Kurita et al., 2016; Ma & Nie, 2019; Nitta et al., 2014). However, while multimodal imaging represents an interesting option opening new possibilities, there are undoubtedly many challenges associated with multimodal approaches (Masyuko et al., 2013). For instance, some challenges are inherent in experimental sample preparation that has to be compatible with all the combined techniques (Buchberger et al., 2018). Another issue is related to the alignment of molecular images recorded independently by different analytical methods, and characterized by image distortions and dissimilar spatial resolutions (in the (*x,y*) plane) but also different depths of penetration (*z* axis) (Patterson et al., 2019; Piqueras et al., 2018). The combined use of multiple analytical modalities therefore requires the implementation of advanced and specialized chemometrics tools. In particular, such chemometric approaches have to be able to merge the datasets generated by orthogonal analytical techniques and to precisely align the images acquired on separate instruments, while considering the specificity of all techniques (Ahlf et al., 2014; Buchberger et al., 2018). In this context, multiblock methods are particularly valuable as they are able to simultaneously evaluate multiple complex and large datasets obtained from different modalities, combined into one single model (Bedia et al., 2020; Nikitina et al., 2020).

This review summarizes the analytical strategies and current applications of SALDI‐MS imaging. Yet, further research for technical improvements, mechanistic understanding, and innovative SALDI‐MSI approaches are expected in the near future as the SALDI‐MS technique is gaining interest since the early 2000′s. In particular, the complementarity of SALDI‐MSI with MALDI‐MSI will have to be more deeply exploited to maximize the molecular information extracted from complex samples. Certainly, the development of targeted approaches as well as multimodal methodologies will also open new opportunities, notably in the detection of low abundant/ionizable compounds. We are convinced that SALDI‐MSI will play a key role in the forthcoming years in addressing the crucial needs for molecular imaging of small molecules, with improved analytical performance.

## CONFLICTS OF INTEREST

The authors declare that there are no conflicts of interest.
